# MicroRNA-200c-targeted contactin 1 facilitates the replication of influenza A virus by accelerating the degradation of MAVS

**DOI:** 10.1371/journal.ppat.1010299

**Published:** 2022-02-16

**Authors:** Shuai Xu, Lu Han, Yanli Wei, Bo Zhang, Qian Wang, Junwen Liu, Minxuan Liu, Zhaoshan Chen, Zhengxiang Wang, Hualan Chen, Qiyun Zhu

**Affiliations:** 1 State Key Laboratory of Veterinary Etiological Biology, College of Animal Medicine, Lanzhou University, Lanzhou Veterinary Research Institute, Chinese Academy of Agricultural Sciences, Lanzhou, PR China; 2 State Key Laboratory of Veterinary Biotechnology, Harbin Veterinary Research Institute, Chinese Academy of Agricultural Sciences, Harbin, PR China; Emory University School of Medicine, UNITED STATES

## Abstract

Influenza A viruses (IAVs) continuously challenge the poultry industry and human health. Elucidation of the host factors that modulate the IAV lifecycle is vital for developing antiviral drugs and vaccines. In this study, we infected A549 cells with IAVs and found that host protein contactin-1 (CNTN1), a member of the immunoglobulin superfamily, enhanced viral replication. Bioinformatic prediction and experimental validation indicated that the expression of CNTN1 was reduced by microRNA-200c (miR-200c) through directly targeting. We further showed that CNTN1-modulated viral replication in A549 cells is dependent on type I interferon signaling. Co-immunoprecipitation experiments revealed that CNTN1 specifically interacts with MAVS and promotes its proteasomal degradation by removing its K63-linked ubiquitination. Moreover, we discovered that the deubiquitinase USP25 is recruited by CNTN1 to catalyze the deubiquitination of K63-linked MAVS. Consequently, the CNTN1-induced degradation cascade of MAVS blocked RIG-I-MAVS-mediated interferon signaling, leading to enhanced viral replication. Taken together, our data reveal novel roles of CNTN1 in the type I interferon pathway and regulatory mechanism of IAV replication.

## Author summary

Type I interferon is an essential component of host immunity against influenza A virus infection. However, the regulatory mechanisms involved in the IAV-induced type I interferon pathway are not fully elucidated. Here, we identified CNTN1 as a critical host factor for the replication of IAV. CNTN1 promotes viral replication by negatively regulating the RIG-I-mediated type I interferon pathway. Mechanistically, CNTN1 specifically enhances the proteasomal degradation of MAVS by promoting USP25-mediated deubiquitination. Our findings uncover novel roles of the host protein CNTN1 in type I interferon signaling and IAV replication.

## Introduction

Influenza A viruses (IAVs) are segmented, single-stranded, negative-sense RNA viruses. They are divided into 18 HA and 11 NA subtypes based on the antigenicity of their HA and NA surface glycoproteins. IAVs continuously challenge the poultry industry and human health due to antigenic shift and drift. In the 20th century, H1N1, H2N2, and H3N2 viruses caused four influenza pandemics in humans, resulting in widespread disease and severe loss of human life [1,2]. Since 2003, H5N1 IAVs have infected more than 800 individuals across 16 countries, with an overall case fatality rate of 53% [3,4]. Other H5Nx viruses, such as H5N6 and H5N8, have been detected in wild birds and domestic poultry in many countries, and caused human infections and even deaths [5–7]. The H7N9 low pathogenic avian influenza viruses identified in 2013, and the subsequently mutated H7N9 highly pathogenic avian influenza viruses in 2017, led to 1568 human infections, including 615 fatal cases [8,9]. Therefore, studies on the factors and mechanisms underlying the host-virus interaction remain essential for developing novel anti-influenza therapies.

Innate immunity is the first line of host defense. Interferons, as vital part of the innate immune system, play important roles against invading pathogens during the early stages of infection. Upon IAV infection, the host pattern recognition receptors (PRRs) sense the invading viral RNA and initiate a cascade of signaling responses [10]. Influenza viral RNA is generally sensed by retinoic acid-inducible gene-I (RIG-I) and melanoma differentiation-associated gene 5 (MDA5). RIG-I undergoes conformational changes and translocation to the mitochondrial membrane, where it interacts with the mitochondrially localized adaptor protein MAVS (also known as VISA, IPS-1, and Cardif), thereby recruiting downstream effector molecules and activating IRF3, IRF7, and nuclear factor κB (NF-κB) to produce type I interferons (IFN-I), ultimately creating an antiviral state [11,12].

In this study, using transcriptome deep-sequencing and data analysis, we discovered that host factor contactin-1 (CNTN1) expression was reduced upon infection with various subtypes of IAV. Further, we found that CNTN1 facilitates the replication of IAV through negative regulation of the RIG-I-mediated IFN-I pathway. Mechanistically, CNTN1 recruits USP25 to deubiquitinate K63-linked MAVS, which promotes the proteasomal degradation of MAVS. Our data reveal a novel mechanism by which CNTN1 negatively regulates RIG-I-mediated IFN-I pathway and potentiates IAV replication.

## Results

### CNTN1 is downregulated by IAV infection and promotes the replication of IAVs in A549 cells

**Fig 1:**
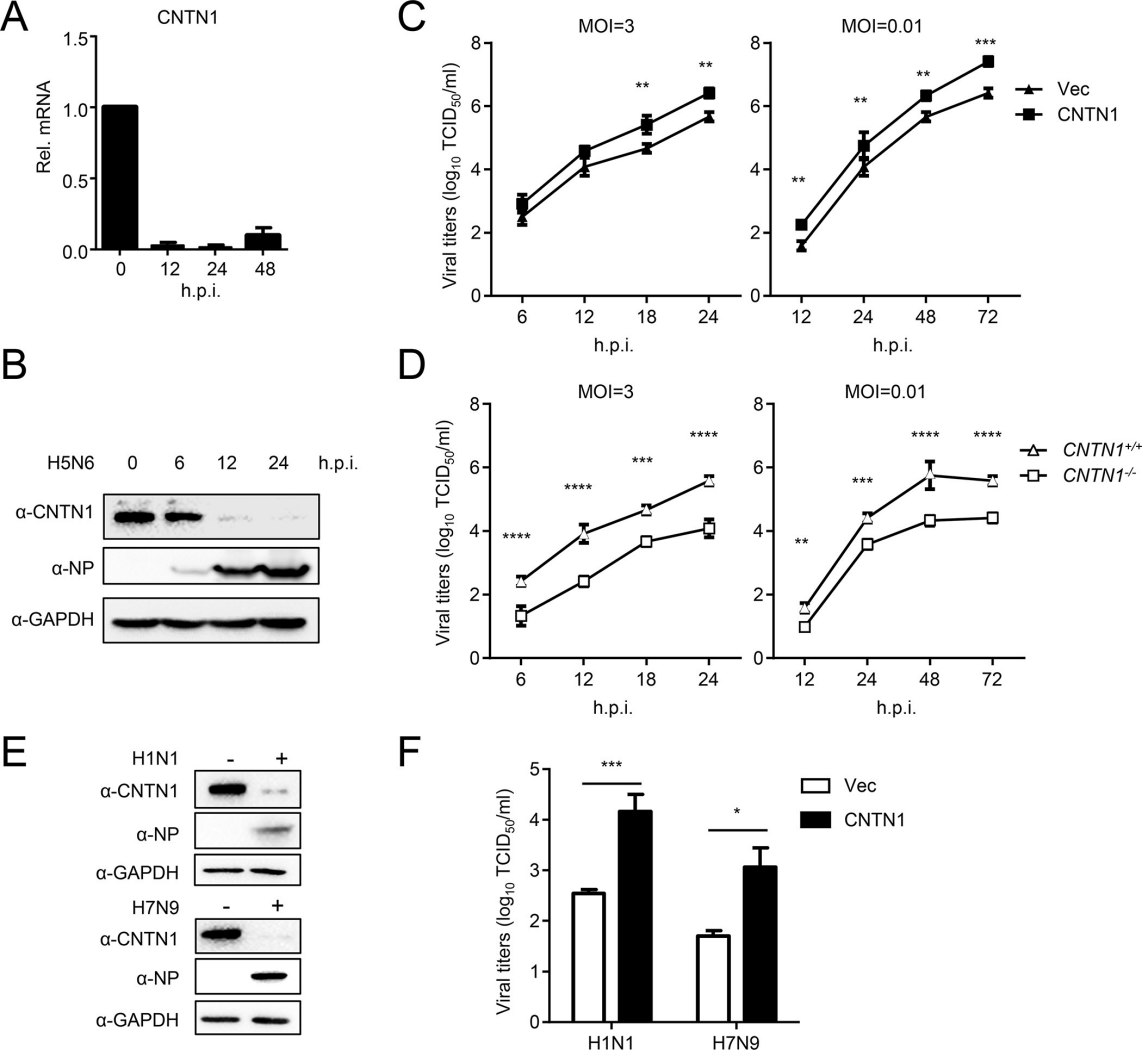
CNTN1 facilitates influenza A virus replication. (A) A549 cells were infected with H5N6 virus (MOI = 1), and the CNTN1 mRNA as quantified at different times post-infection by use of qPCR analysis. (B) A549 cells were infected with H5N6 virus (MOI = 1), and the expression level of CNTN1 protein was quantified at indicated times post-infection. (C) A549 cells were transfected with either the CNTN1 expression plasmid or empty vector (Vec). Twenty-four hours later, the cells were infected with H5N6 virus at an MOI of 0.01 or 3. At the indicated times post-infection, the supernatant containing viral particles was assessed in the TCID_50_ assay. (D) *CNTN1*^*+/+*^ or *CNTN1*^*-/-*^ A549 cells were infected with H5N6 virus at an MOI of 0.01 or 3. At the indicated times post-infection, the supernatant containing viral particles was assessed in the TCID_50_ assay. (E) A549 cells were infected with H1N1 or H7N9 virus (MOI = 1), and the expression level of CNTN1 protein was quantified at 24 h post-infection. (F) A549 cells were transfected with either the CNTN1 expression plasmid or Vec. Twenty-four hours later, the cells were infected with H1N1 or H7N9 virus at an MOI of 1. Twenty-four hours post infection, the supernatant containing viral particles was assessed in the TCID_50_ assay. The data shown represent three independent experiments; bars represent the mean ± SD of the three independent experiments (n = 3). [*P*< 0.05 (*), *P* < 0.01 (**), *P* < 0.001 (***), *P* < 0.0001 (****)].

To screen for host factors involved in the host-virus interaction, we profiled the mRNA expression in A549 cells infected with or without H5N6 virus. We identified groups of genes that were upregulated or downregulated (Log_2_ Fold-change >3, *P-value* <0.05) after H5N6 virus infection (S1 Table). We noticed that one of the IAV-downregulated genes, CNTN1, has not previously been linked to IAVs. We confirmed that CNTN1 was dramatically downregulated by H5N6 virus infection (Fig 1A and 1B). To investigate the effect of virus-downregulated CNTN1 on IAV replication, we used CRISPR/Cas9 to knockout the endogenous CNTN1 in A549 cells (S1A Fig). Viral growth kinetics assays indicated that overexpressed CNTN1 significantly facilitated H5N6 replication, whereas CNTN1-deficiency significantly inhibited viral replication (Fig 1C and 1D). Furthermore, CNTN1 levels were observably decreased in A549 cells following infection with H1N1 or H7N9 viruses (Fig 1E). CNTN1 also facilitated the replication of H1N1 and H7N9 viruses in A549 cells (Fig 1F). These data indicate that virus-downregulated CNTN1 generally promotes the replication of IAVs.

### IAV-induced miR-200c negatively regulates the expression of CNTN1

**Fig 2:**
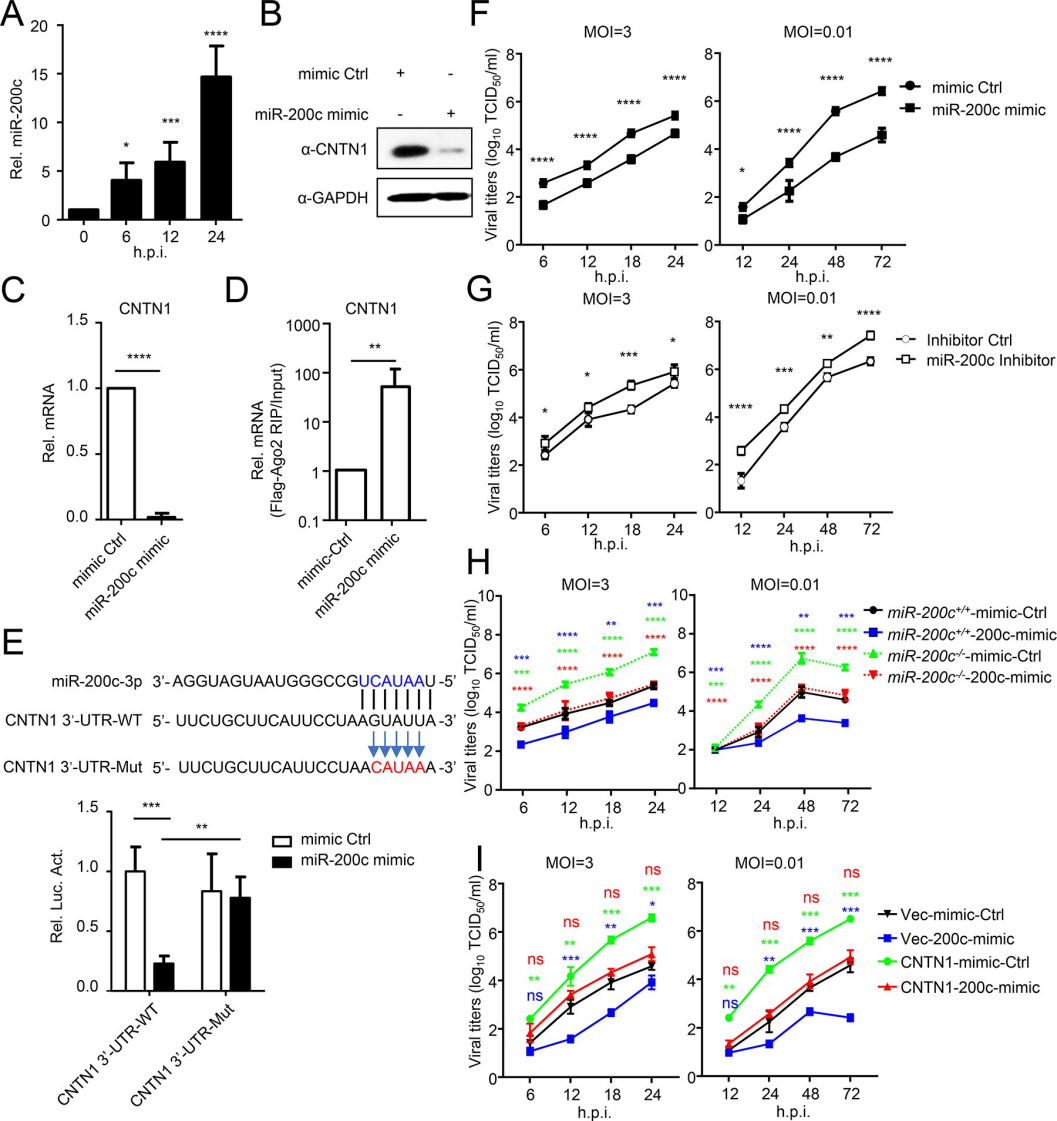
CNTN1 expression is downregulated by virus-induced miR-200c. (A) The expression of miR-200c in A549 cells infected with H5N6 virus for indicated times was quantified by using qPCR analysis. (B-C) HEK293 cells were transfected with miR-200c mimic or mimic control for 24 h before immunoblotting and qPCR analysis. (D) A549 cells were transfected with Flag-Ago2 in the presence of either the miR-200c mimic or mimic control. Twenty-four hours later, the cells were subjected to RIP assay with an anti-Flag antibody. The level of CNTN1 mRNA was quantified by using a qPCR assay. (E) Predicted miR-200c binding sites in the 3’-UTR of the CNTN1 mRNA. Perfect matches in seed regions are indicated by lines. Mutations (indicated by arrows) were generated in the binding sites of the 3’-UTR for the reporter gene assay. HEK293 cells were transfected with the wild-type or mutant of the CNTN1 3’-UTR reporter plasmid and the miR-200c mimic or mimic control for 24 h prior to the luciferase assays. (F) A549 cells were transfected with the miR-200c mimic or mimic control, followed by infection with H5N6 virus at an MOI of 0.01 or 3. At the indicated times post-infection, the supernatant containing viral particles was assessed in the TCID_50_ assay. (G) A549 cells were transfected with the miR-200c inhibitor or inhibitor control, followed by infection with H5N6 virus at an MOI of 0.01 or 3. At the indicated times post-infection, the supernatant containing viral particles was assessed in the TCID_50_ assay. (H) *miR-200c*^*+/+*^ or *miR-200c*^*-/-*^ cells were transfected with the miR-200c mimic or mimic control. Twenty-four hours later, the cells were infected with H5N6 virus at an MOI of 0.01 or 3. At the indicated times post-infection, the supernatant containing viral particles was assessed in the TCID_50_ assay. The data collected from *miR-200c*^*-/-*^ cells are shown as dotted lines. The significant difference between *miR-200c*^*+/+*^-mimic-Ctrl and *miR-200c*^*+/+*^-200c-mimic is labeled in blue; the significant difference between *miR-200c*^*+/+*^-mimic-Ctrl and *miR-200c*^*-/-*^-mimic-Ctrl is labeled in green; the significant difference between *miR-200c*^*-/-*^-mimic-Ctrl and *miR-200c*^*-/-*^-200c-mimic is labeled in red. (I) A549 cells were transfected with the miR-200c mimic or mimic control, and the CNTN1 expression plasmid or Vec. Twenty-four hours later, the cells were infected with H5N6 virus at an MOI of 0.01 or 3. At the indicated times post-infection, the supernatant containing viral particles was assessed in the TCID_50_ assay. The significant difference between Vec-mimic-Ctrl and Vec-200c-mimic is labeled in blue; the significant difference between Vec-mimic-Ctrl and CNTN1-mimic-Ctrl is labeled in green; the significant difference between Vec-mimic-Ctrl and CNTN1-200c-mimic is labeled in red. The data shown represent three independent experiments; bars represent the mean ± SD of the three independent experiments (n = 3). [*P*< 0.05 (*), *P* < 0.01 (**), *P* < 0.001 (***), *P* < 0.0001 (****); ‘ns’ indicates no significant difference].

Within the sequencing data, we found that several miRNAs were significantly up- or down-regulated following H5N6 virus infection (S1 Table). We therefore asked whether there was an endogenous miRNA that can specifically suppresses the expression of CNTN1 and promote the replication of H5N6 virus. To screen the miRNAs that target CNTN1, we used MicroRNA.org [13], TargetScan [14] and StarBase [15] software to predict the miRNA binding sites on the 3’-untranslated region (3’-UTR) of human CNTN1. Based on the predicted results, we screened out miR-200c, which appeared to target the 3’-UTR of CNTN1 mRNA. Of note, miR-200c expression in A549 cells was increased following H5N6 virus infection (Fig 2A). To verify the regulatory effect of miR-200c on CNTN1, miR-200c mimic and inhibitor were used to simulate the expression of miR-200c. As shown in S1B Fig, transfection of the miR-200c mimic and inhibitor had no significant impact on A549 cell viability. However, treatment with the miR-200c mimic markedly reduced CNTN1 expression (Fig 2B). Quantitative PCR (qPCR) revealed that the amount of CNTN1 mRNA was significantly reduced following transfection of the cells with the miR-200c mimic (Fig 2C).

To confirm that the mRNA of CNTN1 directly associated with miR-200c, we first performed an RNA immunoprecipitation (RIP) assay using Flag-Ago2 (a component of the miRNA-silencing complex), and found that the mRNA of CNTN1 was considerably enriched within the miR-200c group (Fig 2D). In addition, we cloned the region of the CNTN1 3’-UTR that contained the predicted target site of miR-200c into the dual-luciferase reporter vector pmirGLO; we also constructed the 3’-UTR region with mutations in the binding site and cloned it into the pmirGLO vector to obtain a mutant plasmid (Fig 2E). HEK293 cells were transfected the wild-type CNTN1 3’-UTR plasmid or mutant plasmid in the presence of the miR-200c mimic or miRNA control for 24 h. The miR-200c mimic significantly inhibited the luciferase activity of the wild-type CNTN1 3’-UTR plasmid-transfected cells, whereas transfection with the mutant CNTN1 3’-UTR abolished the inhibitory effect of the miR-200c mimic on the luciferase activity (Fig 2E), suggesting that miR-200c directly interacts with CNTN1 3’-UTR.

We next addressed the effect of miR-200c on the replication of H5N6 virus. At 24 h after miR-200c mimic transfection, A549 cells were infected with H5N6 virus (MOI = 3 or 0.01). The viral titers of the cell supernatants were measured by TCID_50_ assay, and the results showed that the miR-200c mimic significantly inhibited viral replication (Fig 2F). We also investigated the effect of the miR-200c inhibitor on IAV replication, and found that treatment with the miR-200c inhibitor led to a marked increase in titers (Fig 2G). Additionally, using CRISPR/Cas9 to knockout the endogenous miR-200c in A549 cells, we found that miR-200c-deficiency significantly promoted viral replication, while miR-200c mimic restored the inhibitory effect (Figs S1C and 2H). Furthermore, the inhibitory effect of the miR-200c mimic on viral replication was restored by CNTN1 (Figs 2I and S1D).

Together, our findings indicate that virus-induced miR-200c downregulates CNTN1 expression through direct interaction, thereby inhibiting H5N6 virus replication.

### CNTN1 inhibits type I interferon signaling pathway

**Fig 3:**
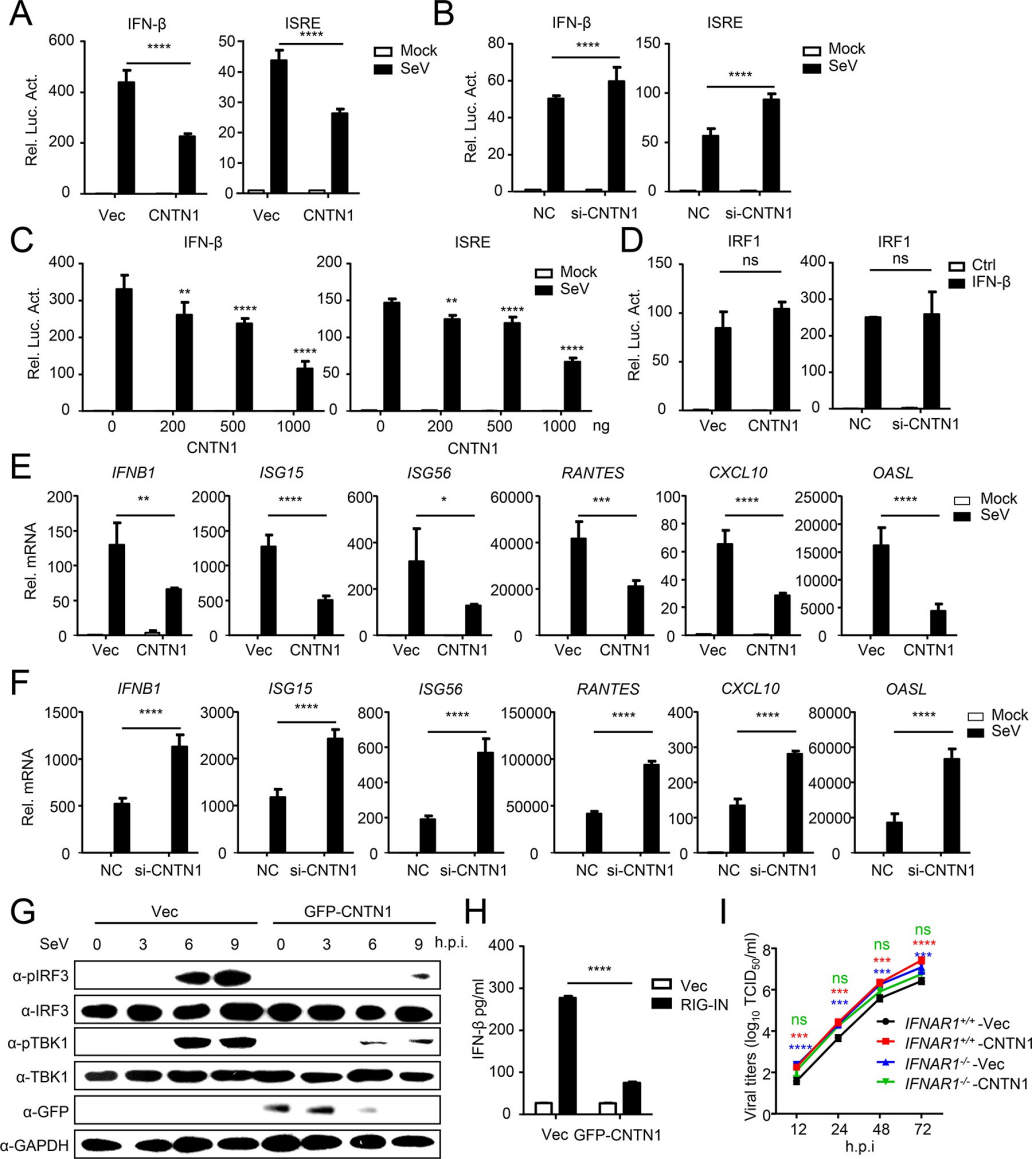
CNTN1 negatively regulates the type I IFN signaling pathway. (A-B) CNTN1 inhibits IFN-related promoter activities. Luciferase reporter plasmids (IFN-β-Luc or ISRE-Luc) and the pRL-TK plasmid were co-transfected into HEK293 cells, along with Flag-CNTN1 or Vec (A) or CNTN1 siRNA or scrambled siRNA (NC) (B). At twenty-four hours after transfection, the cells were left uninfected or were infected with SeV for 12 h before reporter assays. (C) Luciferase reporter plasmids (IFN-β-Luc or ISRE-Luc) and the pRL-TK plasmid were co-transfected into HEK293 cells, along with different concentrations of CNTN1 expression plasmid. At twenty-four hours after transfection, cells were left uninfected or were infected with SeV for 12 h before reporter assays. (D) Luciferase reporter plasmids (IRF1-Luc) and the pRL-TK plasmid were co-transfected into HEK293 cells, along with Flag-CNTN1 or Vec or CNTN1 siRNA or NC. At twenty-four hours after transfection, the cells were left untreated or were treated with IFN-β for 12 h before reporter assays. (E-F) A549 cells were transfected with Flag-CNTN1 or Vec (E) or CNTN1 siRNA or NC (F), and subsequently left uninfected or infected with SeV for 12 h before qPCR analysis. (G) CNTN1 inhibits virus-induced phosphorylation of TBK1 and IRF3. A459 cells were transfected with GFP-CNTN1 plasmid or Vec. At twenty-four hours after transfection, the cells were infected with SeV for the indicated times. Then, immunoblotting analysis were performed with the indicated antibodies. (H) HEK293 cells were transfected with CNTN1 or Vec, and induced by RIG-IN transfection. The concentration of IFN-β in the supernatants was detected by an ELISA. (I) *IFNAR1*^*+/+*^ or *IFNAR1*^*-/-*^ A549 cells were transfected with the CNTN1 expression plasmid or Vec. Twenty-four hours later, the cells were infected with H5N6 virus at an MOI of 0.01 or 3. At the indicated times post-infection, the supernatant containing viral particles was assessed in the TCID_50_ assay. The significant difference between *IFNAR1*^*+/+*^-Vec and *IFNAR1*^*+/+*^-CNTN1 is labeled in red; the significant difference between *IFNAR1*^*+/+*^-Vec and *IFNAR1*^*-/-*^-Vec is labeled in blue; the significant difference between *IFNAR1*^*-/-*^-Vec and *IFNAR1*^*-/-*^-CNTN1 is labeled in green. The data shown represent three independent experiments; bars represent the mean ± SD of the three independent experiments (n = 3). [*P*< 0.05 (*), *P* < 0.01 (**), *P* < 0.001 (***), *P* < 0.0001 (****); ‘ns’ indicates no significant difference].

Upon IAV infection, the RIG-I-mediated IFN-I signaling pathway is the main innate immune response [16]. To investigate the mechanism of CNTN1-promoted IAV replication, we first tested whether CNTN1 affects the activity of the IFN-I signaling pathway. As shown in Fig 3A, overexpressed CNTN1 strongly inhibited Sendai virus (SeV)-triggered activation of the IFN-β promoter and interferon sequence response element (ISRE). When we used siRNA to knock down CNTN1, the cell viability was not markedly influenced and the activation of the IFN-β promoter and ISRE was significantly elevated (Figs S2A and S2B and 3B). In addition, CNTN1 inhibited the activation of the IFN-β promoter in a dose-dependent manner (Fig 3C). In contrast, CNTN1 did not affect the activation of IRF1 or STAT1 triggered by IFN-β (Figs 3D and S2C). Our qPCR analysis indicated that CNTN1 significantly inhibited SeV-triggered transcription of the Interferon beta 1 (*IFNB1)*, IFN-stimulated gene 15 (*ISG15*), IFN-stimulated gene 56 (*ISG56*), Regulated upon activation normal T cell expressed and secreted factor (*RANTES*), C-X-C motif chemokine ligand 10 (*CXCL10*) and Oligoadenylate synthetase-like protein (*OASL*) genes in A549 cells (Fig 3E). CNTN1 knockdown or knockout significantly promoted the expression of *IFNB1* and ISG genes (Figs 3F and S2D). Meanwhile, CNTN1 deficiency significantly promoted IAV-induced expression of ISG15 proteins in A549 cells (S2E Fig). SeV-induced phosphorylation of TBK1 and IRF3 were markedly inhibited by CNTN1, suggesting that CNTN1 blocks the activation of TBK1 and IRF3 (Fig 3G). Consistent with this finding, CNTN1 significantly inhibited the induction of IFN-β triggered by RIG-I-CARD (RIG-IN) (Fig 3H). To confirm that CNTN1-promoted viral replication was dependent on the IFN-I pathway, we used CRISPR/Cas9 to knock out endogenous interferon alpha/beta receptor 1 (IFNAR1) in A549 cells (S2F Fig). Viral growth kinetics assays showed that IFNAR1-deficiency abolished CNTN1-promoted viral replication, suggesting that CNTN1 promotes viral replication in an interferon-dependent manner (Fig 3I).

As miR-200c negatively regulates the expression of CNTN1, the role of miR-200c in the innate immune response was also demonstrated. As shown in S2G Fig, miR-200c deficiency significantly promoted IAV-induced transcription of the *IFNB1* and ISG genes. We further detected the protein level of viral NP and ISG15, the results indicated that miR-200c deficiency potentiated the expression of ISG15, and miR-200c mimic restored the promotion (S2H Fig). Taken together, these results clearly demonstrate the role of miR-200c-CNTN1 axis in the regulation of IFN-I signaling.

Since previous studies demonstrated that CNTN1 participates in multiple signaling pathways [17–20], we further investigated the role of CNTN1 in the TLR signaling and in defense against DNA viral infection. As shown in S2I and S2J Fig, overexpression of CNTN1 inhibited poly(I:C)-triggered activation of the IFN-β promoter in TLR3-expressed HEK293 cells, and promoted the replication of herpes simplex virus 1 (HSV-1), which suggested that CNTN1 participates in the regulation of TLR signaling and innate immune response against DNA virus. Since the RIG-I is considered to be the main viral RNA sensor and readily activated upon IAV infection [16], the present study focused on the role of CNTN1 in the RIG-I-mediated IFN-I signaling pathway.

### CNTN1 specifically interacts with and degrades MAVS

**Fig 4:**
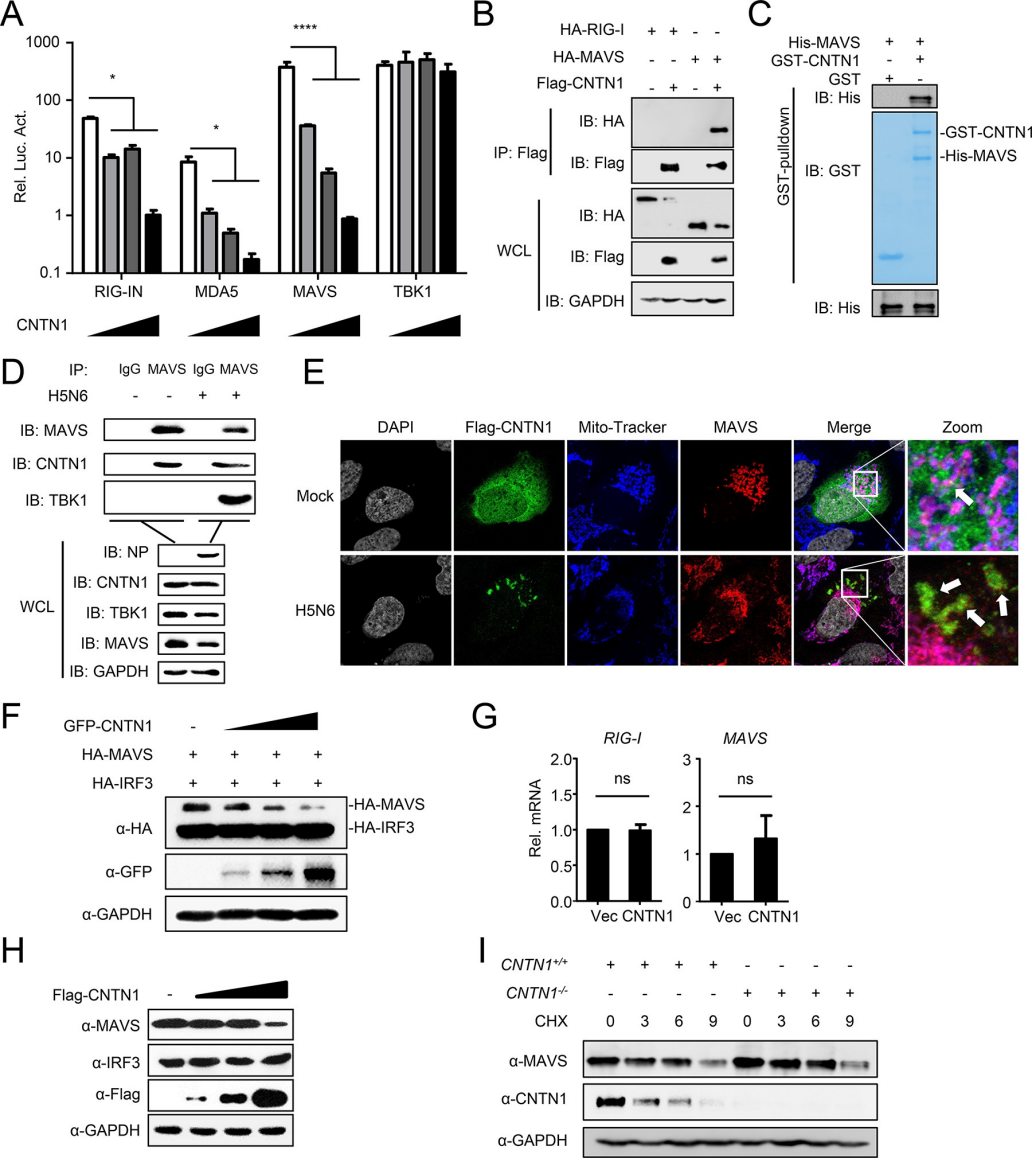
CNTN1 specifically interacts with MAVS and promotes degradation. (A) CNTN1 inhibits activation of the IFN-β promoter induced by RIG-IN, MDA5, and MAVS. HEK293 cells were transfected with the indicated plasmids along with control vector or increasing amounts of CNTN1 expression plasmids. Reporter assays were performed 24 h after transfection. (B) Overexpressed CNTN1 interacts with MAVS. HEK293 cells were transfected with the indicated plasmids for 24 h. Then, co-immunoprecipitation and immunoblotting analysis were performed with the indicated antibodies. (C) CNTN1 interacts with MAVS directly. Purified GST-CNTN1 was used to pull-down purified His-MAVS. (D) Endogenous MAVS interacts with CNTN1. HEK293 cells were uninfected or infected with H5N6 virus for 24 h before co-immunoprecipitation and immunoblotting analysis. (E) CNTN1 colocalizes with MAVS in the cell cytoplasm. U2OS cells were transfected with Flag-CNTN1 plasmid for 24 h, and subsequently left uninfected or infected with IAV for 12 h before staining with mito-tracker. In the zoomed images, the yellow color indicated the colocalization of Flag-CNTN1 and MAVS, the purple color indicated the colocalization of mitochondria and MAVS. (F) Overexpression of CNTN1 decreases the MAVS protein level. HEK293 cells were transfected with HA-MAVS and HA-IRF3 and increasing amounts of Flag-CNTN1 for 24 h before immunoblotting analysis. (G) CNTN1 has no effect on the expression of MAVS mRNA. HEK293 cells were transfected with Vec or the CNTN1 plasmid for 24 h before qPCR analysis. (H) CNTN1 decreases endogenous MAVS expression. HEK293 cells were transfected with increasing amounts of Flag-CNTN1 for 24 h before immunoblotting analysis. (I) *CNTN1*^*+/+*^ and *CNTN1*^*-/-*^ cells were treated with CHX (100 μg/mL). Cells were harvested for total protein extraction at 0, 3, 6, and 9 h after treatment, then immunoblotting analysis was performed with the indicated antibodies. The data shown represent three independent experiments; bars represent the mean ± SD of the three independent experiments (n = 3). [*P*< 0.05 (*), *P* < 0.01 (**), *P* < 0.001 (***), *P* < 0.0001 (****); ‘ns’ indicates no significant difference].

We next investigated how CNTN1 inhibits the IFN-I signaling pathway. To determine the molecular target of CNTN1 in the regulation of virus-triggered IFN-I activation, we transfected plasmids encoding RIG-IN, MDA5, MAVS, or TBK1 together with the IFN-β promoter in the presence or absence of CNTN1. CNTN1 inhibited the activation of the IFN-β promoter triggered by the expression of RIG-IN, MDA5, and MAVS in a dose-dependent manner, but not TBK1 (Fig 4A). Co-immunoprecipitation (Co-IP) experiments indicated that MAVS, but not RIG-I, specifically interacted with CNTN1 (Fig 4B). Pull-down assays showed that GST-CNTN1 directly interacted with His-MAVS *in vitro* (Fig 4C). Endogenous Co-IP experiments further verified that endogenous CNTN1 directly associates with MAVS (Fig 4D). Confocal microscopy confirmed that MAVS was mostly localized on mitochondria and CNTN1 dispersed throughout the cytoplasm in cells without IAV infection, which showed little colocalization with MAVS. While CNTN1 protein was downregulated and congregated in the cytoplasm in the IAV-infected cells, and colocalized with partial MAVS outside of mitochondria (Fig 4E).

CNTN1 protein is composed of six C2 immunoglobulin-like domains, four fibronectin type III (FNIII) domains, and a glycosylphosphatidylinositol (GPI)-linkage (S3A Fig) [21]. We constructed four CNTN1 truncations as shown in S3A Fig. The results of domain mapping experiments indicated that all truncations can immunoprecipitated MAVS (S3B and S3C Fig). The data suggest that both the Ig-like domains and FNIII domains are the critical domains for the interaction with MAVS.

Because MAVS expression was reduced in CNTN1-overexpressing cells (Fig 4B), we hypothesized that CNTN1 might regulates the expression or stability of MAVS. To test this hypothesis, we co-transfected CNTN1 together with HA-tagged MAVS and IRF3 and performed immunoblotting analysis. CNTN1 specifically downregulated the expression of MAVS, but not that of IRF3 (Fig 4F). Next, we assessed the transcription of MAVS in A549 cells, and found that CNTN1 did not affect the transcription of MAVS (Fig 4G). Of note, overexpressed CNTN1 downregulated the expression of endogenous MAVS, but not IRF3 (Fig 4H). Additionally, the transfection experiments in the wild-type and CNTN1-deficient cells treated with CHX indicated that CNTN1 deficiency prolonged the half-life of endogenous MAVS (Fig 4I).

Collectively, our results reveal that CNTN1 specifically interacts with and induces the degradation of MAVS.

### CNTN1 promotes the degradation of MAVS through a ubiquitin-proteasomal pathway

**Fig 5:**
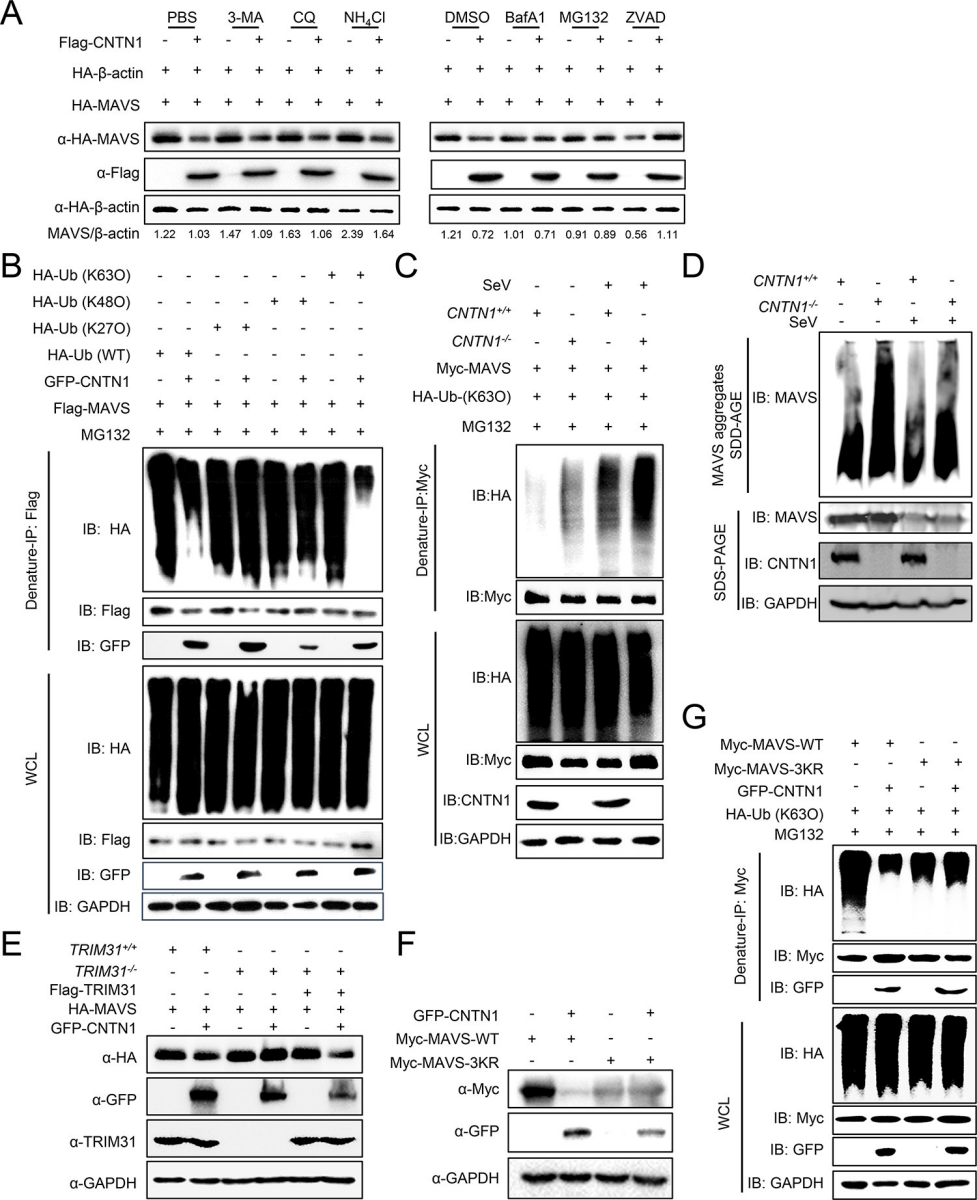
CNTN1 promotes proteasomal degradation of MAVS. (A) MG132 and ZVAD blocks CNTN1-mediated MAVS degradation. HEK293 cells were transfected with the indicated plasmids for 20 h and then treated with 3-MA (10 mM), CQ (50 μM), NH_4_Cl (20 mM), MG132 (10 μM), BafA1 (0.2 μM), or ZVAD (20 μM) for 6 h. The cell lysates were then analyzed by immunoblotting with the indicated antibodies. The intensities of the indicated protein bands were determined by image J, normalized to HA-β-actin, and are shown as the fold-change of MAVS/β-actin. (B) CNTN1 inhibits K63-linked ubiquitination of MAVS. HEK293 cells transfected with Flag-MAVS, HA-ubiquitin, or its mutants [KO, in which all but one of the lysine residues were simultaneously mutated to arginine (K-only)], together with a control or CNTN1 plasmids, were pre-treated with MG132 for 6 h. The cells were then subjected to denature-immunoprecipitation (denature-IP) and immunoblotting analysis with the indicated antibodies. (C) *CNTN1*^*+/+*^ and *CNTN1*^*-/-*^ cells were transfected with Myc-MAVS, HA-ubiquitin (K63O), and were left uninfected or infected with SeV for 12 h. The cells were then subjected to denature-IP and immunoblotting analysis with the indicated antibodies. (D) *CNTN1*^*+/+*^ and *CNTN1*^*-/-*^ cells were left uninfected or infected with SeV for 12 h. Twenty-four hours later, cells were harvested for SDD-AGE or SDS-PAGE followed by immunoblotting analysis with the indicated antibodies. (E) TRIM31 deficiency inhibits CNTN1 mediated degradation of MAVS. Wild-type HEK293 cells or TRIM31-deficient cells were transfected with the indicated plasmids for 24 h, followed by immunoblotting analysis with the indicated antibodies. (F) HEK293 cells transfected with GFP-CNTN1 and wild-type MAVS or the MAVS 3KR mutant (K11, 311, and 461R) for 24 h before immunoblotting analysis. (G) CNTN1 does not inhibit K63-linked ubiquitination of the MAVS 3KR mutant. HEK293 cells transfected with wild-type MAVS or the MAVS 3KR mutant, HA-ubiquitin (K63O), together with a control or CNTN1 plasmids, were pre-treated with MG132 (10 μM) for 6 h. The cells were then subjected to denature-IP and immunoblotting analysis with the indicated antibodies.

The ubiquitin-proteasome pathway and the autolysosome pathway are the major intracellular protein degradation pathways [22]. To determine which of these pathways is responsible for CNTN1-mediated MAVS degradation, we added the proteasome inhibitor MG132, the caspase inhibitor ZVAD, and the autophagy inhibitors 3-methylademine (3-MA), chloroquine (CQ), and bafilomycin A1 (Baf A1), or the lysosome inhibitor ammonium chloride (NH_4_Cl) to cells co-transfected with CNTN1 and MAVS. CNTN1-mediated MAVS degradation was mostly restored by treatment with MG132 or ZVAD, suggesting that CNTN1 mediates the degradation of MAVS in a proteasome-dependent manner (Fig 5A).

Ubiquitin chains attached to substrates serve as a major signal for proteasomal degradation [23]. By using a ubiquitination assay, we found that K48- and K27-polyubiquitination of MAVS was not affected by CNTN1; however, K63-polyubiquitination of MAVS was severely impaired in the presence of CNTN1 (Fig 5B), suggesting that CNTN1 might promotes proteasomal degradation of MAVS by attenuating K63-linked ubiquitination. Conversely, in CNTN1-deficient cells, the K63-linked deubiquitination of MAVS was markedly increased with or without SeV stimulation (Fig 5C). It was reported that K63 ubiquitination contributed to the prion-like aggregates of MAVS [24]. The semi-denaturing detergent agarose-gel electrophoresis (SDD–AGE) assay indicated that the aggregation of MAVS was higher in *CNTN1*^*−/−*^ cells than that in *CNTN1*^*+/+*^ cells (Fig 5D).

Since TRIM31 is the only reported E3 ubiquitin ligase, catalyzing the K63-linked polyubiquitination of MAVS at lysine residues 11, 311, and 461 [25], we hypothesized that TRIM31-deficiency or MAVS with K11R, K311R, and K461R mutations (MAVS-3KR) may prevent CNTN1-mediated K63-polyubiquitination-dependent degradation. As expected, CNTN1-mediated degradation of MAVS was restored in *TRIM31*^*-/-*^ cells (Fig 5E). Moreover, MAVS-3KR largely impaired the K63-linked polyubiquitination of MAVS, and blocked the CNTN1-reduced K63-polyubiquitination of MAVS, leading to the abolished degradation of MAVS-3KR (Fig 5F and 5G). These results show that CNTN1 promotes proteasomal degradation of MAVS by attenuating the K63-linked ubiquitination of MAVS.

### CNTN1 potentiates USP25-mediated proteasomal degradation of MAVS

**Fig 6:**
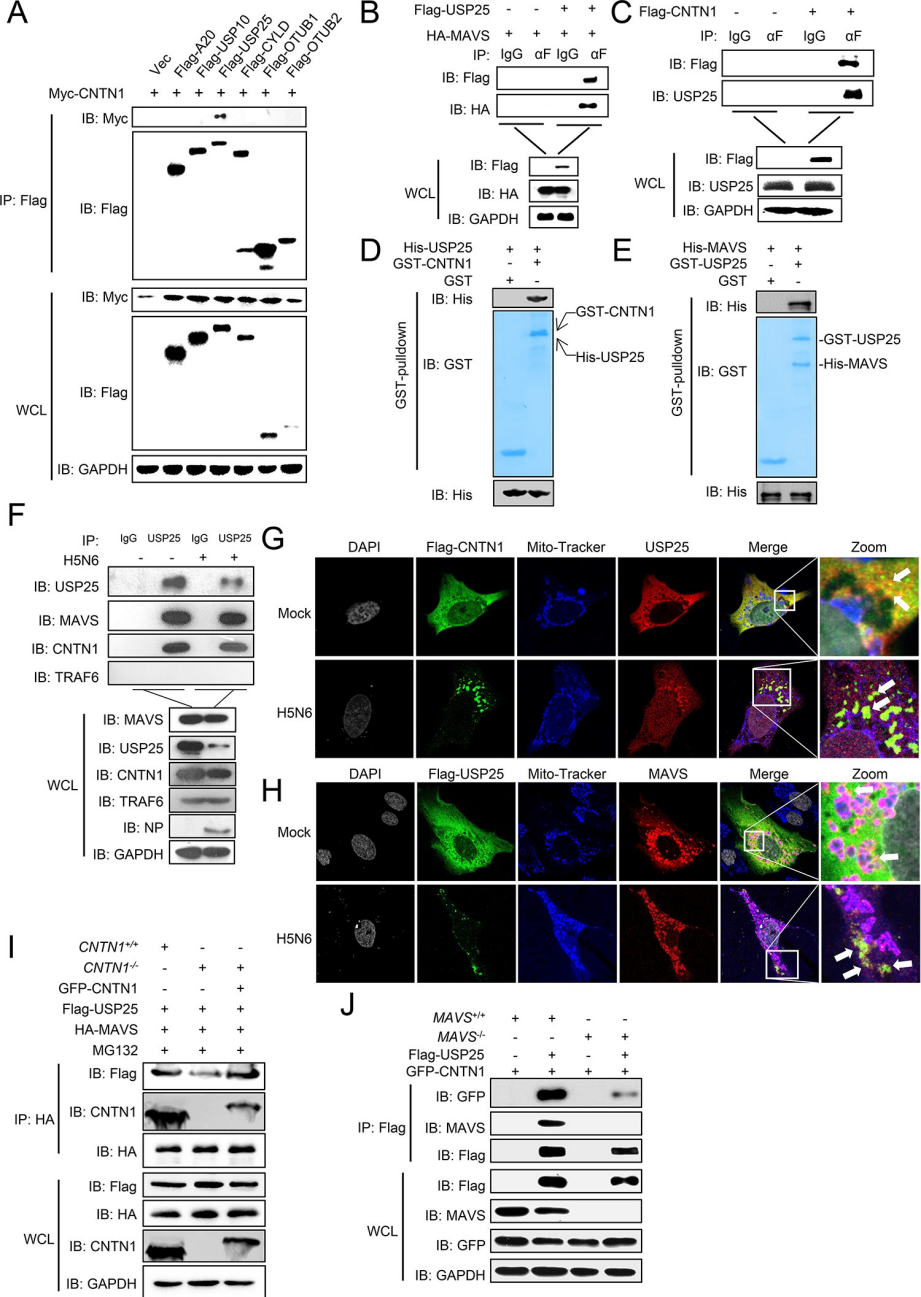
CNTN1 specifically interacts with USP25. (A) Overexpressed CNTN1 interacts with USP25. HEK293 cells were transfected with the indicated plasmids for 24 h before co-immunoprecipitation and immunoblotting analysis. (B) Overexpressed USP25 interacts with HA-MAVS. HEK293 cells were transfected with the indicated plasmids for 24 h before co-immunoprecipitation and immunoblotting analysis. (C) Endogenous USP25 interacts with CNTN1. HEK293 cells were transfected with the indicated plasmids for 24 h before co-immunoprecipitation and immunoblotting analysis. (D) USP25 interacts with MAVS directly. Purified GST-USP25 was used to pull-down purified His-MAVS. (E) CNTN1 interacts with USP25 directly. Purified GST-CNTN1 was used to pull-down purified His-USP25. (F) Endogenous MAVS interacts with USP25. HEK293 cells were left uninfected or infected with H5N6 virus for 24 h before co-immunoprecipitation and immunoblotting analysis. (G) CNTN1 colocalizes with USP25 in the cell cytoplasm. U2OS cells were transfected with a Flag-CNTN1 plasmid for 24 h, and subsequently left uninfected or infected with H5N6 virus for 12 h before staining with mito-tracker. In the zoomed images, the yellow color indicated the colocalization of Flag-CNTN1 and USP25. (H) USP25 colocalizes with MAVS in the cell cytoplasm. U2OS cells were transfected with a Flag-USP25 plasmid for 24 h, and subsequently left uninfected or infected with H5N6 virus for 12 h before staining with mito-tracker. In the zoomed images, the yellow color indicated the colocalization of Flag-USP25 and MAVS, the purple color indicated the colocalization of mitochondria and MAVS. (I) CNTN1-deficiency inhibits the interaction between USP25 and MAVS. *CNTN1*^*+/+*^ or *CNTN1*^*-/-*^ HEK293 cells were transfected with the indicated plasmids for 24 h before co-immunoprecipitation and immunoblotting analysis. (J) MAVS-deficiency inhibits the interaction between USP25 and CNTN1. *MAVS*^*+/+*^ or *MAVS*^*-/-*^ HEK293 cells were transfected with the indicated plasmids for 24 h before co-immunoprecipitation and immunoblotting analysis.

CNTN1 is neither an E3-ubiquitin ligase nor a deubiquitination enzyme (DUB). Therefore, we hypothesized that CNTN1 might functions as a scaffold to link DUBs to MAVS for deubiquitination and degradation. Previous studies have identified several DUBs that participate in the antiviral response, including A20, USP10, USP25, CYLD, OTUB1, and OTUB2 [26–28]. Co-IP experiments revealed that CNTN1 specifically interacts with USP25, but not other DUBs (Fig 6A). Flag-USP25 specifically interacted with HA-MAVS, and endogenous USP25 interacted with Flag-CNTN1 (Fig 6B and 6C). Pull-down analysis showed that His-MAVS directly interacts with GST-USP25, and His-USP25 directly interacts with GST-CNTN1 (Fig 6D and 6E). As expected, endogenous USP25 specifically interacted with MAVS and CNTN1 (Fig 6F). Domain mapping experiments suggest that the Ig-like domains of CNTN1 are critical for the interaction with USP25 (S3D Fig).

To determine the colocalization of CNTN1, USP25, and MAVS in infected or uninfected cells, the immunofluorescent experiment was conducted. Confocal microscopy showed that CNTN1 and USP25 colocalized and dispersed throughout the cytoplasm without virus infection, which had a partial association with MAVS. While USP25 protein was downregulated and congregated with CNTN1 in the cytoplasm of the IAV-infected cells, and colocalized with partial of MAVS that did not associate with mitochondria (Fig 6G and 6H). Moreover, the interaction between USP25 and MAVS was significantly impaired in CNTN1-deficient cells (Fig 6I), and CNTN1-USP25 interaction was markedly abrogated in MAVS-deficient cells (Fig 6J).

It has been reported that miR-200c directly binds to the 3’-UTR of USP25, reducing both the mRNA and protein levels of USP25 [29]. As verification, we found that treatment with the miR-200c mimic markedly reduced USP25 expression (S4A Fig). RNA immunoprecipitation assay showed that the mRNA of USP25 was considerably enriched within the miR-200c group (S4B Fig). Since miR-200c was upregulated following virus infection, we confirmed the role of miR-200c in the regulation of CNTN1 and USP25 in miR-200c-deficient cells. The results indicated that CNTN1 and USP25 were downregulated by H5N6 virus infection, while the virus-mediated decrease of CNTN1 and USP25 was restored in miR-200c-deficient cells, suggesting that miR-200c downregulates CNTN1 and USP25 expression through direct interactions (S4C Fig).

**Fig 7:**
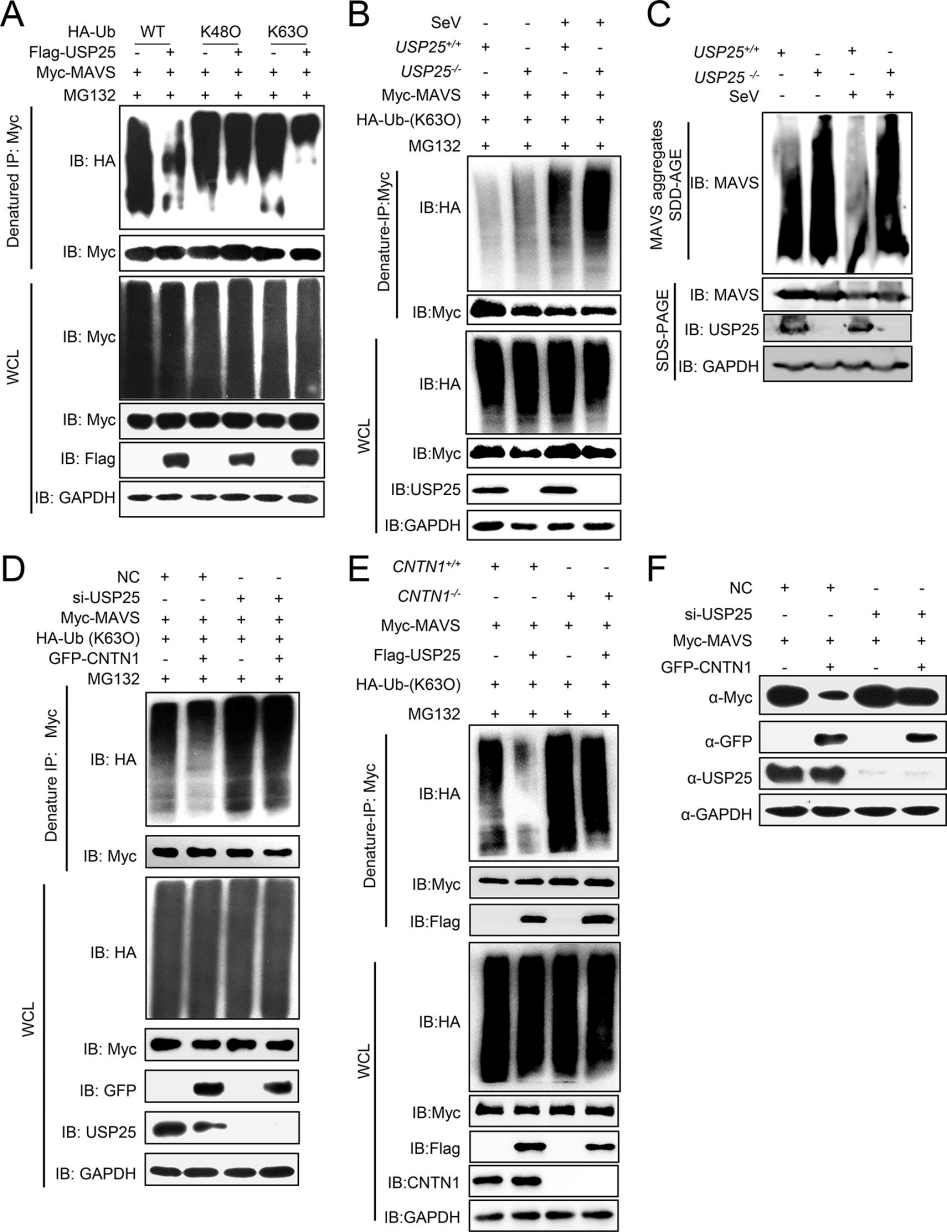
CNTN1 facilitates USP25 to degrade MAVS through the proteasomal pathway. (A) USP25 inhibits K63-linked ubiquitination of MAVS. HEK293 cells transfected with Myc-MAVS, HA-ubiquitin, or its mutants (K48O, K63O), together with a control or USP25 plasmids, were pre-treated with MG132 (10 μM) for 6 h, and the cells were then subjected to denature-IP and immunoblotting analysis with the indicated antibodies. (B) *USP25*^*+/+*^ and *USP25*^*-/-*^ cells transfected with Myc-MAVS, HA-ubiquitin (K63O), were left uninfected or infected with SeV for 12 h. The cells were then subjected to denature-IP and immunoblotting analysis with the indicated antibodies. (C) *USP25*^*+/+*^ and *USP25*^*-/-*^ cells were left uninfected or infected with SeV for 12 h. Twenty-four hours later, cells were harvested for SDD-AGE or SDS-PAGE followed by immunoblotting analysis with the indicated antibodies. (D) USP25-knockdown inhibits CNTN1-triggered K63-linked ubiquitination of MAVS. HEK293 cells were transfected with the indicated plasmids and USP25 siRNA or NC for 24 h. The cells were then treated with MG132 for 6 h before being used in ubiquitination assays with the indicated antibodies. (E) *CNTN1*^*+/+*^ or *CNTN1*^*-/-*^ HEK293 cells were transfected with Myc-MAVS and HA-ubiquitin (K63O), together with a control or USP25 plasmids. The cells were then subjected to denature-IP and immunoblotting analysis with the indicated antibodies. (F) USP25-knockdown inhibits CNTN1-mediated degradation of MAVS. HEK293 cells were transfected with the indicated plasmids and USP25 siRNA or NC for 24 h before immunoblotting analysis.

Next, we determined whether and how USP25 mediates the K63-linked deubiquitination and degradation of MAVS. Using the ubiquitination assay, we found that USP25 catalyzed the K63-linked deubiquitination of MAVS (Fig 7A). The K63-linked deubiquitination of MAVS was markedly increased in the USP25-deficient cells infected or uninfected with SeV (Figs 7B and S4D). The SDD–AGE assay indicated that USP25-deficiency increased the aggregation of MAVS, while the CNTN1-induced K63-linked deubiquitination of MAVS was markedly reverted in the USP25-knockdown cells (Fig 7C and 7D). Furthermore, the USP25-catalyzed K63-linked deubiquitination of MAVS was mostly restored in the CNTN1-deficient cells (Fig 7E). These data suggest that CNTN1 recruits USP25 to mediate K63-linked deubiquitination of MAVS. We further revealed that knockdown of USP25 inhibits CNTN1-mediated degradation of MAVS (Fig 7F).

Together, our results show that CNTN1 potentiates USP25-mediated K63-linked deubiquitination and proteasomal degradation of MAVS.

## Discussion

In this study, we demonstrated that endogenous CNTN1 is downregulated by virus-induced miR-200c. CNTN1 acts as a host suppressor of innate immunity by mediating the degradation of MAVS. CNTN1 collaborates with the deubiquitinating enzyme USP25 to mediate K63-linked deubiquitination of MAVS for proteasomal degradation. We have thus uncovered a novel mechanism through which the host factor CNTN1 promotes the replication of influenza A viruses through suppression of innate immune responses.

CNTN1 is the first identified member of the contactin family within the immunoglobulin superfamily, and a GPI-anchored membrane protein that facilitates cell adhesion. CNTN1 and other members of the CNTN family share a common structure of six C2 Ig-like repeats, four FNIII domains, and a GPI-linkage at the C-terminus that anchors the protein to the extracellular plasma membrane [21]. CNTN1 is highly expressed in the human brain and neuronal tissues, and plays an essential role in nervous system development, including neural cell adhesion, myelination, neurite growth, axonal elongation, and fasciculation [30–32]. Recent reports have revealed that CNTN1 is also involved in carcinogenesis and cancer progression [17–19]. For example, CNTN1 serves as a downstream effector of the vascular endothelial growth factor C (VEGFC)/FLT4 signaling pathway, which activates SRC/p38 mitogen-activated protein kinase (MAPK)-CEBPA signaling [19]. CNTN1 also promotes epithelial-mesenchymal transition, by activating the PI3K/AKT signaling pathway [20]. Nevertheless, the molecular mechanism of CNTN1 involved host-virus interaction has never been demonstrated, although one study described that CNTN1 potentially interacted with classical swine fever virus p7 [33]. The present study discovered that CNTN1 is downregulated upon infection with H1N1, H5N6, or H7N9 IAV subtypes. In addition, CNTN1 facilitated the replication of these viruses (Fig 1). CNTN1 thus participates in the regulatory mechanism of IAV replication.

The innate immune response is the first line of defense against viral infection, and RIG-I-mediated type I interferon signaling is one of the most important innate immune responses against infection with RNA viruses, such as influenza A virus [34]. Upon influenza A virus infection, type I IFNs are secreted and bind to the cognate receptor, type I IFN receptor (IFNAR), to initiate a signaling cascade that leads to the transcriptional induction of various ISGs [35]. Our results showed that IFNAR1-knockout abolished the CNTN1-mediated facilitation of IAV replication (Fig 3), demonstrating that CNTN1 facilitates IAV replication by suppressing the innate immune responses. We further revealed that CNTN1 inhibits the RIG-I-mediated IFN-I signaling pathway and decreases the expression of IFN-β and IFN-stimulated genes, therefore promotes the replication of IAVs (Fig 3). Additionally, we confirmed that CNTN1 plays roles in the TLR signaling pathway and in defense against HSV-1 infection, which should be further investigated (S2I and S2J Fig).

As a mitochondrial membrane protein, MAVS functions as a critical adaptor of the RLR signaling pathway that links upstream recognition of viral RNA to downstream signal activation during antiviral responses [12]. Mounting evidence suggests that MAVS may be a common target for the regulation of the IFN-I signaling pathway. For viruses, certain viral proteins exert negative effects of innate immunity by targeting MAVS for immune evasion [36–40]. For example, Li *et al*. revealed that the nonstructural protein 3 of Zika virus interacts with and induces degradation of MAVS to evade antiviral responses of host cells [41]. One of our previous studies revealed that the PB1 protein of H7N9 virus recruits the selective autophagic receptor NBR1 to associate with MAVS for autophagic degradation, resulting in disrupted innate signaling and enhanced viral replication [42]. For host cells, the activity of MAVS should be finely tuned to exert sufficient protective immune responses while avoiding excessive harmful immune pathology. Numerous factors have been reported to be involved in this fine-tuning. RNF115 constitutively interacts with MAVS and induces proteasomal degradation of MAVS in uninfected cells [43]. The Golgi protein 73 (GP73) interacts with MAVS and promotes its degradation to negatively regulate innate immunity [44]. Here, we revealed that CNTN1 inhibits IFN-I induction by specifically mediating the degradation of MAVS (Fig 4).

Ubiquitination is strategically positioned to fine-tune the magnitude and duration of immune signaling cascades and, therefore, is also an attractive target for viral subversion mechanisms [45]. Several studies have demonstrated that MAVS could be modified by ubiquitination during viral infection [25,46–49]. Generally, the stability of MAVS is regulated via K48-linked ubiquitination by several ubiquitin E3 ligases (e.g., RNF5, RNF125, AIP4/ITCH, Smurf1/2, and MARCH5), which may control excessive immune responses or be exploited by viruses to block the activation of innate signaling pathways [50–53]. We previously reported that the PB1 protein of H7N9 virus markedly enhances the K27-linked ubiquitination of MAVS to autophagic degradation [42]. However, in the present study, CNTN1 markedly reduced the K63-linked ubiquitination of MAVS for proteasomal degradation (Fig 5). Consistent with this latter finding, Cheung *et al*. demonstrated that the viral protein PB1-F2 of avian influenza virus suppresses K63-polyubiquitination and aggregation of MAVS, and directs unaggregated MAVS for degradation [54].

Following viral infection, MAVS undergoes a conformational switch that leads to the formation of prion-like functional aggregates on the mitochondrion. It is now known that TRIM31-mediated K63-polyubiquitination of MAVS is essential for the aggregation and activation [25]. However, persistent MAVS signaling leads to host immunopathology, and it remains largely unknown how these MAVS aggregates are resolved. RACK1 has been reported to enhances K48-linked ubiquitination of MAVS, attenuates its K63-linked ubiquitination, and decreases MAVS-mediated antiviral signal transduction [55]. Recently, Liu *et al*. found that the deubiquitinase YOD1 cleaves the K63-linked ubiquitination of MAVS and negatively regulates antiviral innate immunity [56]. In the present study, we discovered that the deubiquitinase USP25 is recruited and interacts with CNTN1 to remove K63-linked ubiquitination of MAVS for degradation (Fig 7).

There is accumulating evidence that noncoding RNAs, including circRNAs, lncRNAs, and miRNAs that are key post-transcriptional regulators, and play important roles in virus-host interactions [57–59]. MiR-200c, a well-established tumor suppressor miRNA, participates in the responses against multiple viruses, including hepatitis B virus and hepatitis C virus, by targeting various transcripts [60,61]. One study indicates that H5N1 avian influenza virus upregulates the expression of miR-200c, which reduces the level of Angiotensin-converting enzyme 2 (ACE2), and eventually leads to increased angiotensin II levels and subsequent lung injury [62]. In this study, we showed that miR-200c is upregulated in response to H5N6 virus infection and suppresses viral replication by directly targeting CNTN1 and USP25 to enhance IFN-I signaling (Figs 2 and S4). Of note, the data in this study showed that miR-200c-deficiency did not completely restore the virus-induced degradation of CNTN1 (S4C Fig). Additionally, virus infection led to degradation of GFP-CNTN1 which did not have a miR-200c-targeted 3’UTR (Figs 3G and S4E). Therefore, it’s reasonable to speculate that another certain degradation mechanism, but not miR-200c targeted, was induced to downregulate CNTN1 protein upon virus infection. The underpinned mechanism should be further investigated.

**Fig 8:**
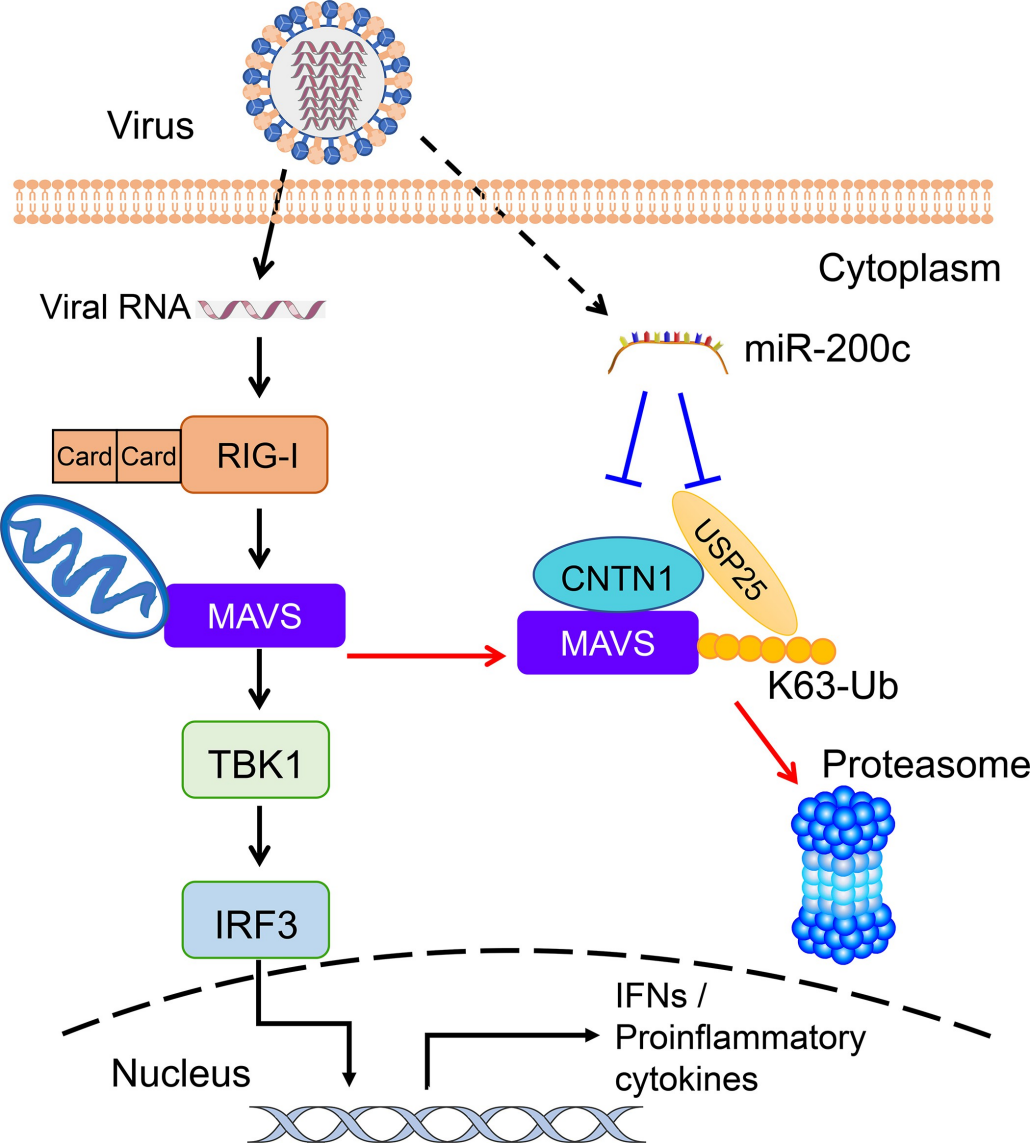
A working model of the role of CNTN1 in the regulation of MAVS-mediated signaling and influenza A virus replication. Black arrows indicate the RIG-I/MAVS-mediated type I IFN signaling pathway. The dotted line indicates the induction of miR-200c following virus infection. The blue lines indicate the interaction between miR-200c and CNTN1/USP25. The red arrows indicate the process of CNTN1-mediated proteasomal degradation of MAVS.

Based on our data, we propose a working model for the regulatory role of CNTN1 in the RIG-I-mediated interferon signaling pathway (Fig 8). In non-infected cells, the CNTN1 protein acts as a negative regulator of the IFN-I pathway, by recruiting USP25 to deubiquitinate the K63-linked MAVS and promoting its degradation. Upon infection by influenza A viruses, the RIG-I-mediated signaling pathway is activated by the viral RNA. To fulfill the needs of type I interferon production, miR-200c is upregulated to reduce CNTN1 and USP25 expression and inhibit MAVS degradation. Finally, the RIG-I-mediated innate signaling is elevated, leading to the promotion of type I IFN responses and suppression of viral replication. Our findings clarify the role of CNTN1 in immune function against IAVs and provide valuable insights into the MAVS regulation network.

## Materials and methods

### Biosafety and ethical statements

This study was performed in strict accordance with the recommendations in the Guide for the Care and Use of Laboratory Animals of the Ministry of Science and Technology of the People’s Republic of China. Studies with highly pathogenic H5N6 avian influenza viruses were carried out in a biosecurity level 3 laboratory approved for such use by the Chinese Ministry of Agriculture and Rural Affairs. The protocol was approved by the Committee on the Ethics of Animal Experiments of the Harbin Veterinary Research Institute (HVRI) of the Chinese Academy of Agricultural Sciences (CAAS). The details of the facility and the biosafety and biosecurity measures used have been previously reported [63].

### Cells, viruses, and plasmids

MDCK, HEK293 and U2OS cells were grown in DMEM (Gibco) supplemented with 10% (vol/vol) FBS (Gibco-BRL; 10099–141) and 1× penicillin/streptomycin (Gibco-BRL; 10378016). A549 cells were grown in Kaighn’s modified Ham F-12 nutrient mixture medium (Gibco) supplemented with 10% FBS and penicillin/streptomycin. All cells were cultured and maintained at 37°C with 5% CO_2_.

For *CNTN1*^*-/-*^, *USP25*^*-/-*^, *IFNAR1*^*-/-*^, *TRIM31*^*-/-*^ and *miR-200c*^*-/-*^ cells, target sequences were cloned into pX459 digested with BpiI. Transfected HEK293/A549 cells were purified by puromycin selection. *MAVS*^*-/-*^ cells were kindly provided by Prof. Xin Cao (Jilin Agricultural University, China).

H5N6 influenza A virus (A/duck/Guangdong/S1330/2016, GD/330) was isolated in Guangdong Province in China in 2016 [63]. H1N1 influenza virus (A/Sichuan/1/2009) was isolated from the first human case of the 2009 influenza pandemic in China. H7N9 virus (A/Suzhou/SZ19/2014) was isolated from Jiangsu Province in China in 2014 [42]. Sendai virus (SeV) was kindly provided by Hongkui Deng (Peking University, China). HSV-1 was kindly provided by Bo Zhong (Wuhan University, China). Virus stocks were propagated in specific-pathogen-free (SPF) chicken eggs and stored at -70°C until use.

The wild-type (WT) and mutant 3’-UTR of human CNTN1 was synthesized by Tsingke (China) and inserted into the pmirGLO Dual-Luciferase miRNA Target Expression Vector (Promega). Human CNTN1, TRIM31, CNTN1 truncations, and Argonaute2 were cloned from the cDNA of A549 cells into the pRK-Flag vector by using standard molecular biology techniques. Plasmids for HA-, Flag-, and Myc-tagged CNTN1, RIG-I, MAVS, TRAF3, TBK1, IKKε, IRF3, MAVS mutants (K10, 311, 461R) and ubiquitin (HA-ubiquitin or its mutants) [64], and use of the IFN-β-Luc, ISRE-Luc, IRF1-Luc, STAT1-Luc, and pRL-TK internal control luciferase reporter plasmids used in the study were described previously [65]. Plasmids for Flag-tagged A20, USP10, CYLD, OTUB1, OTUB2, USP25, and GAPDH were kindly provided by Dr. Bo Zhong (Wuhan University, China). His-tagged MAVS, USP25, and CNTN1 were constructed and cloned into the pET-28a prokaryotic expression vector, and GST-tagged USP25 and CNTN1 were constructed and cloned into the pGEX-4T-1 prokaryotic expression vector by using standard molecular biology techniques.

### Reagents and antibodies

The antibodies used in this study were as follows: poly(I:C) (tlrl-picwlv, InvivoGen); anti-CNTN1 (AF904, R&D); anti-GFP (11814460001), HRP-conjugated anti-HA (12013819001), anti-Myc (11814150001) antibodies (Roche); MAVS (24930S), TRAF6 (8028S), TBK1 (3013S), IRF3 (4302S), phosphorylated TBK1(5483), IRF3 (4961) antibodies (Cell Signaling Technology); anti-GST-Tag (M20007L) and HRP-conjugated goat anti-mouse secondary antibody (M21001L) (Abmart); anti-TRIM31 (12543) antibodies (Proteinteck); anti-His (TA-02) and HRP-conjugated goat anti-rabbit IgG (ZB-2301) (Zsbio); anti-IFNAR1 antibody (bs-4116R, Bioss); anti-GAPDH (ab181602), anti-USP25 (ab246948), anti-ISG15 (ab285367) antibodies (Abcam); Alexa Fluor 488-conjugated anti-mouse IgG (A0428), Alexa Fluor 647-conjugated goat anti-rabbit IgG (A0468) secondary antibodies (Beyotime); anti-NP (11675-MM03T) antibodies (Sino Biological); and HRP-conjugated anti-Flag (A8592) antibodies (Sigma).

Reagents used in the study included: 3-MA (M9281), MG132 (M7449), DMSO (D2650), CQ (PHR1258), NH_4_Cl (09718), anti-Flag agarose affinity beads (A2220) and protein A/G agarose affinity beads (P6486/E3403) (Sigma); CHX (ab120093, Abcam); human IFN-β DuoSet ELISA kit (R&D, DY814-05); Glutathione Sepharose 4B (17-0756-05, GE Healthcare); Baf-A1 (S1314, Selleck,); ZVAD (C1202), NP-40 (ST366), DAPI (C1002), Mito-Tracker Red CMXRos (C1049B), recombinant Human IFN-β (P5660) (Beyotime). The scrambled negative control RNA (NC), miR-200c mimic and inhibitor, and CNTN1-specific short interfering RNA (siRNA, 5’-GCGGAAGGTTCTAGAACCA-3’) were purchased from RiboBio Co. (China). Lipofectamine 2000, RNAi MAX and TRIzol were obtained from Invitrogen (USA). SYBR Green I Master Mix was purchased from Roche (Germany).

### RNA isolation and quantitative PCR

Total RNA from cells was extracted with TRIzol following the manufacturer’s instructions. For mRNAs, total RNA was subsequently transcribed into cDNA using M-MLV Reverse Transcriptase, according to the manufacturer’s protocol (Promega). For miRNAs, total RNA was reverse-transcribed according to the protocol provided with the All-in-One miRNA First-Strand cDNA Synthesis Kit (Genecopoeia, China). RNU6 and GAPDH were used as invariant controls for miRNAs and mRNAs, respectively. Primers for miR-200c were designed by Genecopoeia Co. (China). Real-time PCR was carried out using the ABI 7500 detection System (Applied Biosystems, USA). The RNA level of each gene is shown as fold of induction (2^-ΔΔCT^) in the graphs. The sequences of the gene-specific primers used for qPCR are provided in S2 Table.

### Viral infection

All cells were seeded at the desired density in culture plates as per the requirements for different experiments. Viruses were inoculated into cells at a specific multiplicity of infection (MOI) for various experiments. One hour after inoculation, the medium was replaced with fresh OPTI-MEM and the cells were incubated at 37°C. Virus-containing culture supernatants were collected at the indicated time points for titration.

### Virus titration

Viral titers of virus stocks and cell culture supernatants were determined by end-point titration in MDCK cells. Ten-fold serial dilutions of each sample were inoculated into MDCK cells. Two days after inoculation, supernatant from the inoculated cells was collected and tested for the ability to agglutinate chicken erythrocytes as an indicator of viral replication. Infectious viral titers are reported as log_10_ TCID_50_/mL, and were calculated from 3 replicates by using the method of Reed–Muench [66].

### CRISPR-Cas9 knockout

Genome engineering was performed using the CRISPR-Cas9 system [67,68]. Double-stranded oligonucleotides corresponding to the target sequences were cloned into the pX459-V2.0-SpCas9-HF1 vector. Two micrograms of each pX459 plasmid containing one of the targeting sequences were simultaneously transfected into A549 cells. The transfected cells were selected with puromycin (1 μg/ml) for at least 7 days to obtain knockout cell pools. The following sequences were targeted for human CNTN1, IFNAR1, USP25, TRIM31 or miR-200c genes:

CNTN1: 5’-TCTGGATAAATGGTATTGAT-3’, 5’-CATTTATCCAGAGGAATCAC-3’, 5’-AAAAGTCTCACTCAACTGTA-3’; IFNAR1: 5’-GCGGCTGCGGACAACACCCA-3’, 5’-GACCCTAGTGCTCGTCGCCG-3’, 5’-CTGCGGCGGCTCCCAGATGA-3’; USP25: 5’-CGCCGCGGGGGCCATGACCG-3’, 5’-CACGTTCTGCTCCACGGTCA-3’, 5’-CTGCAGCACGTTCTGCTCCA-3’; TRIM31: 5’-GCCAGTAAATCCGTGATAGC-3’, 5’-GATGTTCCACTATTTCTGCG-3’; miR-200c: 5’-TCGTCTTACCCAGCAGTGTT-3’, 5’-TGGGAGTCTCTAATACTGCC-3’.

### Prediction of miRNA targeting the 3’-UTR of the CNTN1 mRNA

The miRNA targeting the 3’-UTR of CNTN1 mRNA was predicted and selected using MicroRNA.org [13], TargetScan [14] and starBase [15]. All analysis was performed by considering the best optimum scores of each program.

### Dual-luciferase reporter assays

To validate the miRNA targeting CNTN1, luciferase reporter vectors containing WT CNTN1 3’-UTR or the mutant were co-transfected with the control mimic or miR-200c mimic into HEK293 cells. At twenty-four hours after transfection, the transfected cell lysates were analyzed by using the dual luciferase assay kit (Promega). All obtained luciferase values were normalized against those of the Renilla luciferase control.

To detect activation of the IFN-I pathway, HEK293 cells grown in 24-well plates were co-transfected with Luciferase reporter plasmids (IFN-β-Luc, ISRE-Luc, or STAT1-Luc) and the pRL-TK plasmid, along with the indicated amount of empty vector or plasmids expressing CNTN1 or other molecules. At 24 h post-transfection, the cells were left untreated or were treated with SeV, IFN-β, or IAV for an additional 12 h. Cell lysates were prepared and analyzed for firefly and Renilla luciferase activities by using the dual luciferase assay kit (Promega).

### RIP assay

RIP assay was performed as described previously [69]. Briefly, HEK293 cells were lysed in 0.5% NP-40, 150 mM KCl, 25 mM tris-glycine (pH 7.5) and incubated with M2 Flag affinity beads overnight. The lysate was then washed with 300 mM NaCl, 50 mM tris-glycine (pH 7.5), 5 mM MgCl_2_, and 0.05% NP-40. RNA was extracted from the immunoprecipitated RNA-proteins by using the TRIzol reagent according to the manufacturer’s protocol.

### Western blotting

Cells were lysed in RIPA buffer (Beyotime, China). Proteins were separated by 10% SDS-PAGE and transferred to a nitrocellulose membrane (Bio-Rad). The membrane was blocked for 1 h in TBST containing 5% milk and subsequently incubated with primary antibodies overnight at 4°C. After a 1-h incubation with HRP-conjugated secondary antibody, the immunoreactive bands were visualized by using an ECL system (GE Healthcare). The intensities of the target bands were quantified by using the Image J program (NIH, USA).

### GST pull-down assay

GST pull-down assays were conducted as previously described with slight modifications [39]. Briefly, the encoded GST- or His-tagged fusion proteins and the control GST proteins were expressed in BL21 cells after induction with 0.1 mmol/L IPTG overnight at 18°C. Centrifuged cells were resuspended in lysis buffer (1 × PBS, 0.2 mM PMSF, 1% Triton X-100) and sonicated for 15 min. After centrifugation, the supernatant was applied to a Glutathione–Sepharose 4B bead column (GE Healthcare) or ProteinIso Ni-NTA Resin (TransGen Biotech, China), in accordance with the manufacturers’ instructions. Purified GST/His-tagged fusion proteins were diluted with 1× PBS and filtered through Amicon Ultra 0.5 ml filters (Millipore). Then, 1 μg of purified GST protein or GST fusion protein was captured by the Glutathione–Sepharose 4B beads (GE Healthcare), and His-tagged fusion protein was added for incubation overnight at 4°C. The beads were then washed three times with ice-cold PBS. The supernatant was loaded onto gels, followed by immunoblotting analysis.

### Co-immunoprecipitation

HEK293 cells or A549 cells were co-transfected with the indicated plasmids with or without virus infection for 24 h. The transfected cells were then harvested and lysed in NP-40 lysis buffer [20 mM Tris-HCl (pH 7.5), 150 mM NaCl, 1% NP-40, 1 mM EDTA with protease inhibitor cocktails]. For each immunoprecipitation, 1 ml of lysate was incubated for 4 h at 4°C with 0.5 μg of the indicated antibody or control IgG and 30 μL of protein A/G-Sepharose (Sigma). The beads were washed three times with 1 ml of lysis buffer containing 500 mM NaCl. The precipitates were analyzed by using standard immunoblotting procedures.

### Confocal microscopy

Confocal microscopy was performed as previously described [39]. Cells were seeded in 12-well plates (5 × 10^5^ cells/well) on coverslips. At twenty-four hours after transfection, the cells were left uninfected or were infected with IAV (MOI = 1) for 12 h before staining with mito-Tracker (Beyotime). Cells were then fixed with 4% paraformaldehyde for 20 min at room temperature, and washed three times with PBS. Cells were permeabilized with 0.1% Triton X-100 in PBS for 10 min and blocked with 5% skimmed milk for 1 h. Then, the cells were incubated with the indicated primary and secondary antibodies and DAPI. The stained cells were observed with a Leica microscope (TCS SP8) with a 100× oil objective.

### Detection of ubiquitin-modified proteins

The experiments were performed as previously described [70]. Briefly, the cells were lysed in lysis buffer containing 1% SDS and denatured by heating at 95°C for 10 min. After centrifugation, the supernatants were diluted with NP-40 lysis buffer until the concentration of SDS was 0.1%, and were then co-immunoprecipitated with the indicated antibodies. Ubiquitin-modified proteins were detected by immunoblotting with the indicated antibodies.

### SDD-AGE assay

SDD-AGE was performed as previously described [24,54]. Briefly, cells were lysed with cold SDD-AGE lysis buffer (20 mM Tris-HCl pH 8.0, 137 mM NaCl, 10% glycerol, 1% NP-40, 2 mM EDTA and 1× protease inhibitor) for 30 min. Appropriate amount of 5× SDD-AGE sample buffer (2.5× TBE, 2.5% SDS, 25% glycerol and 0.25% bromophenol blue) was then added to SDD-AGE lysate to reach 1×. The crude sample was passed through 25G needle 10 times at 4°C to break viscosity. The SDD-AGE protein sample was then separated by 2% agarose gel and transferred onto PVDF Membrane (BioRad). The membrane was blocked with 5% skimmed milk and then incubated with primary and secondary antibodies. The immunoreactive bands were visualized by using an ECL system (GE Healthcare).

### Statistical analysis

Data are presented as the mean ± SD unless otherwise indicated. Student’s t test was used for all statistical analysis with the GraphPad Prism 6 software (GraphPad Software, USA). Differences between groups were considered significant when the *P-*value was < 0.05 (*), < 0.01 (**), < 0.001 (***), and < 0.0001 (****).

## Supporting information

S1 Fig The effect of CNTN1 or miR-200c knockout. (A) The expression of CNTN1 in *CNTN1*^*+/+*^ and *CNTN1*^*-/-*^ cells. (B) HEK293 cells were transfected with miR-200c mimic, mimic control, miR-200c inhibitor, or inhibitor control. Twenty-four hours later, cell viability was measured by CCK8. (C) The expression of miR-200c in *miR-200c*^*+/+*^ and *miR-200c*^*-/-*^ cells. (D) A549 cells were transfected with the miR-200c mimic or mimic control, and the CNTN1 expression plasmid or Vec. Twenty-four hours later, the cells were infected with H5N6 virus at an MOI of 3. At the 12 hours post-infection, the cells were subjected to immunoblotting analysis with the indicated antibodies.
(TIF)

S2 Fig miR-200c-CNTN1 axis regulates type I interferon signaling pathway. (A) The expression of CNTN1 in NC- or si-CNTN1-transfected cells. (B) A549 cells were transfected with CNTN1 siRNA or NC. At twenty-four hours after transfection, cell viability was measured by CCK8. (C) Luciferase reporter plasmids (STAT1-Luc) and pRL-TK plasmid were co-transfected into HEK293 cells, along with Flag-CNTN1 or Vec. At twenty-four hours after transfection, the cells were left untreated or were treated with IFN-β for 12 h before reporter assays. (D) *CNTN1*^*+/+*^ and *CNTN1*^*-/-*^ cells were transfected with Flag-CNTN1 or Vec, and subsequently left uninfected or infected with SeV for 12 h before qPCR analysis. (E) *CNTN1*^*+/+*^ and *CNTN1*^*-/-*^ cells were transfected with Flag-CNTN1 or Vec, and subsequently left uninfected or infected with H5N6 virus for 24 h before immunoblotting analysis. (F) The expression of IFNAR1 in *IFNAR1*^*+/+*^ and *IFNAR1*^*-/-*^ cells. (G) *miR-200c*^*+/+*^ and *miR-200c*^*-/-*^ cells were left uninfected or infected with SeV for 12 h before qPCR analysis. (H) *miR-200c*^*+/+*^ and *miR-200c*^*-/-*^ cells were transfected with miR-200c mimic or mimic control, and subsequently left uninfected or infected with H5N6 virus for 12 h before immunoblotting analysis. (I) Effects of CNTN1 on TLR3-mediated signaling. 293-TLR3 cells were transfected with IFN-β reporter and pRL-TK plasmid along with the indicated plasmids for 18 h and then were treated or untreated with poly(I:C) (20 μg/mL) for 12 h before reporter assays. (J) A549 cells were transfected with either the CNTN1 expression plasmid or Vec. Twenty-four hours later, the cells were infected with HSV-1 virus. At 24 h post-infection, the expression of HSV-1 RNA was quantified using qPCR analysis.
(TIF)

S3 Fig The key domain of CNTN1 for the interaction with MAVS or USP25. (A) Schematic representation of the domain organization of CNTN1. (B) CNTN1 interacts with MAVS. HEK293 cells were transfected with the indicated plasmids for 24 h. Then the co-immunoprecipitation and immunoblotting analysis were performed with the indicated antibodies. (C) CNTN1 interacts with purified His-MAVS. HEK293 cells were transfected with the indicated plasmids for 24 h. Then the cell lysate was mixed with purified His-MAVS, and the co-immunoprecipitation and immunoblotting analysis were performed with the indicated antibodies. (D) CNTN1 interacts with USP25. HEK293 cells were transfected with the indicated plasmids for 24 h. Then the co-immunoprecipitation and immunoblotting analysis were performed with the indicated antibodies.
(TIF)

S4 Fig miR-200c directly targets USP25. (A) HEK293 cells were transfected with miR-200c the mimic or mimic control for 24 h before immunoblotting analysis. (B) A549 cells were transfected with Flag-Ago2 in the presence of either the miR-200c mimic or mimic control. Twenty-four hours later, the cells were subjected to RIP assay with an anti-Flag antibody. The level of USP25 mRNA was quantified by qPCR. (C) *miR-200c*^*+/+*^ and *miR-200c*^*-/-*^ cells were transfected with GFP-CNTN1 plasmid or Vec. Twenty-four hours later, the cells were infected with H5N6 virus (MOI = 1) for indicated times before immunoblotting analysis. (D) The expression of USP25 in *USP25*^*+/+*^ and *USP25*^*-/-*^ cells. (E) A549 cells were transfected with GFP-CNTN1 plasmid. Twenty-four hours later, the cells were infected with H5N6 virus (MOI = 1) for indicated times post-infection.
(TIF)

S1 Table Differential cellular gene expression in uninfected cells and cells infected with H5N6 virus. (XLSX)

S2 Table PCR primers used in this study. (XLSX)

## References

[1] Medina RA, Garcia-Sastre A. Influenza A viruses: new research developments. Nat Rev Microbiol. 2011 Jul 11;9(8):590–603. doi: 10.1038/nrmicro2613 . 21747392 PMC10433403

[2] Lipsitch M. Avian influenza: Ferret H7N9 flu model questioned. Nature. 2013 Sep 5;501(7465):33. doi: 10.1038/501033e . 24005404

[3] WHO. Cumulative number of confirmed human cases for avian influenza A(H5N1) reported to WHO, 2003–2021, 22 June 2021. 2021.

[4] Song Y, Huang H, Hu Y, Zhang J, Li F, Yin X, et al. A genome-wide CRISPR/Cas9 gene knockout screen identifies immunoglobulin superfamily DCC subclass member 4 as a key host factor that promotes influenza virus endocytosis. PLoS Pathog. 2021 Dec;17(12):e1010141. doi: 10.1371/journal.ppat.1010141 . Pubmed Central PMCID: 8675923. 34871331 PMC8675923

[5] Cui P, Zeng X, Li X, Li Y, Shi J, Zhao C, et al. Genetic and biological characteristics of the globally circulating H5N8 avian influenza viruses and the protective efficacy offered by the poultry vaccine currently used in China. Sci China Life Sci. 2021 2021/11/08. doi: 10.1007/s11427-021-2025-y 34757542

[6] Cui Y, Li Y, Li M, Zhao L, Wang D, Tian J, et al. Evolution and extensive reassortment of H5 influenza viruses isolated from wild birds in China over the past decade. Emerg Microbes Infect. 2020 Dec;9(1):1793–803. doi: 10.1080/22221751.2020.1797542 . Pubmed Central PMCID: PMC7473172. Epub 2020/07/21. 32686602 PMC7473172

[7] Li X, Fu Y, Yang J, Guo J, He J, Guo J, et al. Genetic and biological characterization of two novel reassortant H5N6 swine influenza viruses in mice and chickens. Infection, Infect Genet Evol. 2015 Dec;36:462–6. doi: 10.1016/j.meegid.2015.08.017 . 26296602

[8] Shi J, Deng G, Ma S, Zeng X, Yin X, Li M, et al. Rapid evolution of H7N9 highly pathogenic viruses that emerged in China in 2017. Cell Host & Microbe. 2018 Oct 10;24(4):558–68 e7. doi: 10.1016/j.chom.2018.08.006 . Pubmed Central PMCID: 6310233. 30269969 PMC6310233

[9] Yin X, Deng G, Zeng X, Cui P, Hou Y, Liu Y, et al. Genetic and biological properties of H7N9 avian influenza viruses detected after application of the H7N9 poultry vaccine in China. PLoS Pathog. 2021 Apr;17(4):e1009561. doi: 10.1371/journal.ppat.1009561 . Pubmed Central PMCID: PMC8104392. Epub 2021/04/28. 33905456 PMC8104392

[10] Yoneyama M, Fujita T. RNA recognition and signal transduction by RIG-I-like receptors. Immunol Rev. 2009 Jan;227(1):54–65. doi: 10.1111/j.1600-065X.2008.00727.x . 19120475

[11] Seth RB, Sun L, Ea CK, Chen ZJ. Identification and characterization of MAVS, a mitochondrial antiviral signaling protein that activates NF-kappaB and IRF 3. Cell. 2005 Sep 9;122(5):669–82. doi: 10.1016/j.cell.2005.08.012 . Epub 2005/08/30. 16125763

[12] Xu LG, Wang YY, Han KJ, Li LY, Zhai Z, Shu HB. VISA is an adapter protein required for virus-triggered IFN-beta signaling. Mol Cell. 2005 Sep 16;19(6):727–40. doi: 10.1016/j.molcel.2005.08.014 . Epub 2005/09/13. 16153868

[13] Betel D, Wilson M, Gabow A, Marks DS, Sander C. The microRNA.org resource: targets and expression. Nucleic Acids Res. 2008 Jan;36(Database issue):D149–53. doi: 10.1093/nar/gkm995 . Pubmed Central PMCID: PMC2238905. Epub 2007/12/26. 18158296 PMC2238905

[14] Agarwal V, Bell GW, Nam JW, Bartel DP. Predicting effective microRNA target sites in mammalian mRNAs. Elife. 2015 Aug 12;4. Pubmed Central PMCID: PMC4532895. Epub 2015/08/13. doi: 10.7554/eLife.05005 26267216 PMC4532895

[15] Li JH, Liu S, Zhou H, Qu LH, Yang JH. starBase v2.0: decoding miRNA-ceRNA, miRNA-ncRNA and protein-RNA interaction networks from large-scale CLIP-Seq data. Nucleic Acids Res. 2014 Jan;42(Database issue):D92–7. doi: 10.1093/nar/gkt1248 . Pubmed Central PMCID: PMC3964941. Epub 2013/12/04. 24297251 PMC3964941

[16] Kato H, Takeuchi O, Sato S, Yoneyama M, Yamamoto M, Matsui K, et al. Differential roles of MDA5 and RIG-I helicases in the recognition of RNA viruses. Nature. 2006 May 4;441(7089):101–5. doi: 10.1038/nature04734 . Epub 2006/04/21. 16625202

[17] Chen N, He S, Geng J, Song ZJ, Han PH, Qin J, et al. Overexpression of Contactin 1 promotes growth, migration and invasion in Hs578T breast cancer cells. BMC Cell Biol. 2018 Apr 19;19(1):5. doi: 10.1186/s12860-018-0154-3 . Pubmed Central PMCID: PMC5907708. Epub 2018/04/21. 29673312 PMC5907708

[18] Yan J, Ojo D, Kapoor A, Lin X, Pinthus JH, Aziz T, et al. Neural cell adhesion protein CNTN1 promotes the metastatic progression of prostate cancer. Cancer Res. 2016 Mar 15;76(6):1603–14. doi: 10.1158/0008-5472.CAN-15-1898 . Epub 2016/01/23. 26795349

[19] Su JL, Yang PC, Shih JY, Yang CY, Wei LH, Hsieh CY, et al. The VEGF-C/Flt-4 axis promotes invasion and metastasis of cancer cells. Cancer Cell. 2006 Mar;9(3):209–23. doi: 10.1016/j.ccr.2006.02.018 . Epub 2006/03/15. 16530705

[20] Hung YH, Hung WC. 4-(Methylnitrosamino)-1-(3-pyridyl)-1-butanone (NNK) enhances invasiveness of lung cancer cells by up-regulating contactin-1 via the alpha7 nicotinic acetylcholine receptor/ERK signaling pathway. Chem Biol Interact. 2009 May 15;179(2–3):154–9. doi: 10.1016/j.cbi.2008.10.042 . Epub 2008/11/26. 19027725

[21] Bouyain S, Watkins DJ. The protein tyrosine phosphatases PTPRZ and PTPRG bind to distinct members of the contactin family of neural recognition molecules. Proc Natl Acad Sci U S A. 2010 Feb 9;107(6):2443–8. doi: 10.1073/pnas.0911235107 . Pubmed Central PMCID: PMC2823867. Epub 2010/02/06. 20133774 PMC2823867

[22] Kwon YT, Ciechanover A. The ubiquitin code in the ubiquitin-proteasome system and autophagy. Trends Biochem Sci. 2017 Nov;42(11):873–86. doi: 10.1016/j.tibs.2017.09.002 . Epub 2017/09/28. 28947091

[23] Pohl C, Dikic I. Cellular quality control by the ubiquitin-proteasome system and autophagy. Science. 2019 Nov 15;366(6467):818–22. doi: 10.1126/science.aax3769 . Epub 2019/11/16. 31727826

[24] Hou F, Sun L, Zheng H, Skaug B, Jiang QX, Chen ZJ. MAVS forms functional prion-like aggregates to activate and propagate antiviral innate immune response. Cell. 2011 Aug 5;146(3):448–61. doi: 10.1016/j.cell.2011.06.041 . Pubmed Central PMCID: PMC3179916. Epub 2011/07/26. 21782231 PMC3179916

[25] Liu B, Zhang M, Chu H, Zhang H, Wu H, Song G, et al. The ubiquitin E3 ligase TRIM31 promotes aggregation and activation of the signaling adaptor MAVS through Lys63-linked polyubiquitination. Nat Immunol. 2017 Feb;18(2):214–24. doi: 10.1038/ni.3641 . Epub 2016/12/20. 27992402

[26] Lin D, Zhang M, Zhang MX, Ren Y, Jin J, Zhao Q, et al. Induction of USP25 by viral infection promotes innate antiviral responses by mediating the stabilization of TRAF3 and TRAF6. Proc Natl Acad Sci U S A. 2015 Sep 08;112(36):11324–9. doi: 10.1073/pnas.1509968112 . Pubmed Central PMCID: 4568686. 26305951 PMC4568686

[27] Parvatiyar K, Barber GN, Harhaj EW. TAX1BP1 and A20 inhibit antiviral signaling by targeting TBK1-IKKi kinases. J Biol Chem. 2010 May 14;285(20):14999–5009. doi: 10.1074/jbc.M110.109819 . Pubmed Central PMCID: PMC2865285. Epub 2010/03/23. 20304918 PMC2865285

[28] Jahan AS, Biquand E, Munoz-Moreno R, Le Quang A, Mok CK, Wong HH, et al. OTUB1 is a key regulator of RIG-I-dependent immune signaling and is targeted for proteasomal degradation by influenza A NS1. Cell Rep. 2020 Feb 4;30(5):1570–84 e6. doi: 10.1016/j.celrep.2020.01.015 . Epub 2020/02/06. 32023470

[29] Li J, Tan Q, Yan M, Liu L, Lin H, Zhao F, et al. miRNA-200c inhibits invasion and metastasis of human non-small cell lung cancer by directly targeting ubiquitin specific peptidase 25. Mol Cancer. 2014;13:166. doi: 10.1186/1476-4598-13-166 . Pubmed Central PMCID: PMC4105889. 24997798 PMC4105889

[30] Bizzoca A, Corsi P, Polizzi A, Pinto MF, Xenaki D, Furley AJ, et al. F3/Contactin acts as a modulator of neurogenesis during cerebral cortex development. Dev Biol. 2012 May 1;365(1):133–51. doi: 10.1016/j.ydbio.2012.02.011 . Epub 2012/03/01. 22360968

[31] Mikami T, Yasunaga D, Kitagawa H. Contactin-1 is a functional receptor for neuroregulatory chondroitin sulfate-E. J Biol Chem. 2009 Feb 13;284(7):4494–9. doi: 10.1074/jbc.M809227200 . Epub 2008/12/17. 19075012

[32] Chen YA, Lu IL, Tsai JW. Contactin-1/F3 regulates neuronal migration and morphogenesis through modulating RhoA activity. Front Mol Neurosci. 2018;11:422. doi: 10.3389/fnmol.2018.00422 . Pubmed Central PMCID: PMC6255823. Epub 2018/12/06. 30515076 PMC6255823

[33] Fan J, Zhang M, Liu C, Zhu M, Zhang Z, Wu K, et al. The network of interactions between classical swine fever virus nonstructural protein p7 and host proteins. Front Microbiol. 2020;11:597893. doi: 10.3389/fmicb.2020.597893 . Pubmed Central PMCID: PMC7733924. Epub 2020/12/18. 33329485 PMC7733924

[34] Mibayashi M, Martinez-Sobrido L, Loo YM, Cardenas WB, Gale M Jr., Garcia-Sastre A. Inhibition of retinoic acid-inducible gene I-mediated induction of beta interferon by the NS1 protein of influenza A virus. J Virol. 2007 Jan;81(2):514–24. doi: 10.1128/JVI.01265-06 . Pubmed Central PMCID: PMC1797471. 17079289 PMC1797471

[35] Platanias LC. Mechanisms of type-I- and type-II-interferon-mediated signalling. Nat Rev Immunol. 2005 May;5(5):375–86. doi: 10.1038/nri1604 . Epub 2005/05/03. 15864272

[36] Chen Z, Benureau Y, Rijnbrand R, Yi J, Wang T, Warter L, et al. GB virus B disrupts RIG-I signaling by NS3/4A-mediated cleavage of the adaptor protein MAVS. J Virol. 2007 Jan;81(2):964–76. doi: 10.1128/JVI.02076-06 . Pubmed Central PMCID: 1797450. 17093192 PMC1797450

[37] Wei C, Ni C, Song T, Liu Y, Yang X, Zheng Z, et al. The hepatitis B virus X protein disrupts innate immunity by downregulating mitochondrial antiviral signaling protein. J Immunol. 2010 Jul 15;185(2):1158–68. doi: 10.4049/jimmunol.0903874 . Epub 2010/06/18. 20554965

[38] Wang B, Xi X, Lei X, Zhang X, Cui S, Wang J, et al. Enterovirus 71 protease 2Apro targets MAVS to inhibit anti-viral type I interferon responses. PLoS Pathog. 2013 Mar;9(3):e1003231. doi: 10.1371/journal.ppat.1003231 . Pubmed Central PMCID: PMC3605153. Epub 2013/04/05. 23555247 PMC3605153

[39] Wang J, Lei CQ, Ji Y, Zhou H, Ren Y, Peng Q, et al. Duck tembusu virus nonstructural protein 1 antagonizes IFN-beta signaling pathways by targeting VISA. J Immunol. 2016 Dec 15;197(12):4704–13. doi: 10.4049/jimmunol.1502317 . 27821666

[40] Yoo YS, Park YY, Kim JH, Cho H, Kim SH, Lee HS, et al. The mitochondrial ubiquitin ligase MARCH5 resolves MAVS aggregates during antiviral signalling. Nat Commun. 2015 Aug 6;6:7910. doi: 10.1038/ncomms8910 . Pubmed Central PMCID: 4918326. 26246171 PMC4918326

[41] Li W, Li N, Dai S, Hou G, Guo K, Chen X, et al. Zika virus circumvents host innate immunity by targeting the adaptor proteins MAVS and MITA. FASEB J. 2019 Sep;33(9):9929–44. doi: 10.1096/fj.201900260R . Epub 2019/06/11. 31180720

[42] Zeng Y, Xu S, Wei Y, Zhang X, Wang Q, Jia Y, et al. The PB1 protein of influenza A virus inhibits the innate immune response by targeting MAVS for NBR1-mediated selective autophagic degradation. PLoS Pathog. 2021 Feb;17(2):e1009300. doi: 10.1371/journal.ppat.1009300 . Pubmed Central PMCID: PMC7880438. Epub 2021/02/13. 33577621 PMC7880438

[43] Zhang ZD, Xiong TC, Yao SQ, Wei MC, Chen M, Lin D, et al. RNF115 plays dual roles in innate antiviral responses by catalyzing distinct ubiquitination of MAVS and MITA. Nat Commun. 2020 Nov 2;11(1):5536. doi: 10.1038/s41467-020-19318-3 . Pubmed Central PMCID: PMC7606512. Epub 2020/11/04. 33139700 PMC7606512

[44] Zhang X, Zhu C, Wang T, Jiang H, Ren Y, Zhang Q, et al. GP73 represses host innate immune response to promote virus replication by facilitating MAVS and TRAF6 degradation. PLoS Pathog. 2017 Apr;13(4):e1006321. doi: 10.1371/journal.ppat.1006321 . Pubmed Central PMCID: PMC5398727. Epub 2017/04/11. 28394926 PMC5398727

[45] Ribet D, Cossart P. Pathogen-mediated posttranslational modifications: A re-emerging field. Cell. 2010 Nov 24;143(5):694–702. doi: 10.1016/j.cell.2010.11.019 . Pubmed Central PMCID: PMC7112265. Epub 2010/11/30. 21111231 PMC7112265

[46] Park YJ, Oanh NTK, Heo J, Kim SG, Lee HS, Lee H, et al. Dual targeting of RIG-I and MAVS by MARCH5 mitochondria ubiquitin ligase in innate immunity. Cell Signal. 2020 Mar;67:109520. doi: 10.1016/j.cellsig.2019.109520 . Epub 2019/12/28. 31881323

[47] Liu S, Chen J, Cai X, Wu J, Chen X, Wu YT, et al. MAVS recruits multiple ubiquitin E3 ligases to activate antiviral signaling cascades. Elife. 2013 Aug 14;2:e00785. doi: 10.7554/eLife.00785 . Pubmed Central PMCID: PMC3743401. Epub 2013/08/21. 23951545 PMC3743401

[48] He X, Zhu Y, Zhang Y, Geng Y, Gong J, Geng J, et al. RNF34 functions in immunity and selective mitophagy by targeting MAVS for autophagic degradation. EMBO J. 2019 Jul 15;38(14):e100978. doi: 10.15252/embj.2018100978 . Pubmed Central PMCID: PMC6627233. Epub 2019/07/16. 31304625 PMC6627233

[49] Xue B, Li H, Guo M, Wang J, Xu Y, Zou X, et al. TRIM21 promotes innate immune response to RNA viral infection through lys27-linked polyubiquitination of MAVS. J Virol. 2018 Jul 15;92(14). Pubmed Central PMCID: PMC6026736. Epub 2018/05/11. doi: 10.1128/JVI.00321-18 29743353 PMC6026736

[50] Arimoto K, Takahashi H, Hishiki T, Konishi H, Fujita T, Shimotohno K. Negative regulation of the RIG-I signaling by the ubiquitin ligase RNF125. Proc Natl Acad Sci U S A. 2007 May 1;104(18):7500–5. doi: 10.1073/pnas.0611551104 . Pubmed Central PMCID: PMC1863485. Epub 2007/04/27. 17460044 PMC1863485

[51] You F, Sun H, Zhou X, Sun W, Liang S, Zhai Z, et al. PCBP2 mediates degradation of the adaptor MAVS via the HECT ubiquitin ligase AIP4. Nat Immunol. 2009 Dec;10(12):1300–8. doi: 10.1038/ni.1815 . Epub 2009/11/03. 19881509

[52] Zhong B, Zhang Y, Tan B, Liu TT, Wang YY, Shu HB. The E3 ubiquitin ligase RNF5 targets virus-induced signaling adaptor for ubiquitination and degradation. J Immunol. 2010 Jun 1;184(11):6249–55. doi: 10.4049/jimmunol.0903748 . 20483786

[53] Pan Y, Li R, Meng JL, Mao HT, Zhang Y, Zhang J. Smurf2 negatively modulates RIG-I-dependent antiviral response by targeting VISA/MAVS for ubiquitination and degradation. J Immunol. 2014 May 15;192(10):4758–64. doi: 10.4049/jimmunol.1302632 . Epub 2014/04/15. 24729608

[54] Cheung PH, Lee TT, Kew C, Chen H, Yuen KY, Chan CP, et al. Virus subtype-specific suppression of MAVS aggregation and activation by PB1-F2 protein of influenza A (H7N9) virus. PLoS Pathog. 2020 Jun;16(6):e1008611. doi: 10.1371/journal.ppat.1008611 . Pubmed Central PMCID: PMC7302872. Epub 2020/06/09. 32511263 PMC7302872

[55] Xie T, Chen T, Li C, Wang W, Cao L, Rao H, et al. RACK1 attenuates RLR antiviral signaling by targeting VISA-TRAF complexes. Biochem Biophys Res Commun. 2019 Jan 15;508(3):667–74. doi: 10.1016/j.bbrc.2018.11.203 . Epub 2018/12/12. 30527812

[56] Liu C, Huang S, Wang X, Wen M, Zheng J, Wang W, et al. The otubain YOD1 suppresses aggregation and activation of the signaling adaptor MAVS through Lys63-linked deubiquitination. J Immunol. 2019 May 15;202(10):2957–70. doi: 10.4049/jimmunol.1800656 . Epub 2019/04/07. 30952814

[57] Yang X, Zhao C, Bamunuarachchi G, Wang Y, Liang Y, Huang C, et al. miR-193b represses influenza A virus infection by inhibiting Wnt/beta-catenin signalling. Cell Microbiol. 2019 May;21(5):e13001. doi: 10.1111/cmi.13001 . Pubmed Central PMCID: PMC6459727. Epub 2019/01/17. 30650225 PMC6459727

[58] Sun B, Yang X, Hou F, Yu X, Wang Q, Oh HS, et al. Regulation of host and virus genes by neuronal miR-138 favours herpes simplex virus 1 latency. Nat Microbiol. 2021 May;6(5):682–96. doi: 10.1038/s41564-020-00860-1 . Epub 2021/02/10. 33558653 PMC8221016

[59] Qu Z, Meng F, Shi J, Deng G, Zeng X, Ge J, et al. A novel intronic circular RNA antagonizes influenza virus by absorbing a microRNA that degrades CREBBP and accelerating IFN-beta production. mBio. 2021 Jul 20:e0101721. doi: 10.1128/mBio.01017-21 . Epub 2021/07/21. 34281396 PMC8406138

[60] Tian H, He Z. miR-200c targets nuclear factor IA to suppress HBV replication and gene expression via repressing HBV Enhancer I activity. Biomed Pharmacother. 2018 Mar;99:774–80. doi: 10.1016/j.biopha.2018.01.141 . Epub 2018/05/02. 29710475

[61] Elhelw DS, Riad SE, Shawer H, El-Ekiaby N, Salah A, Zekri A, et al. Ectopic delivery of miR-200c diminishes hepatitis C virus infectivity through transcriptional and translational repression of Occludin. Arch Virol. 2017 Nov;162(11):3283–91. doi: 10.1007/s00705-017-3449-3 . 28642978

[62] Liu Q, Du J, Yu X, Xu J, Huang F, Li X, et al. miRNA-200c-3p is crucial in acute respiratory distress syndrome. Cell Discov. 2017;3:17021. doi: 10.1038/celldisc.2017.21 . Pubmed Central PMCID: PMC5485385. Epub 2017/07/12. 28690868 PMC5485385

[63] Kong X, Guan L, Shi J, Kong H, Zhang Y, Zeng X, et al. A single-amino-acid mutation at position 225 in hemagglutinin attenuates H5N6 influenza virus in mice. Emerg Microbes Infect. 2021 Oct 22:1–21. doi: 10.1080/22221751.2021.1997340 . Epub 2021/10/24. 34686117 PMC8583753

[64] Sun H, Zhang Q, Jing YY, Zhang M, Wang HY, Cai Z, et al. USP13 negatively regulates antiviral responses by deubiquitinating STING. Nat Commun. 2017 May 23;8:15534. doi: 10.1038/ncomms15534 . Pubmed Central PMCID: PMC5457515. Epub 2017/05/24. 28534493 PMC5457515

[65] Wang J, Zeng Y, Xu S, Yang J, Wang W, Zhong B, et al. A naturally occurring deletion in the effector domain of H5N1 swine influenza virus nonstructural protein 1 regulates viral fitness and host innate immunity. J Virol. 2018 Jun 1;92(11). doi: 10.1128/JVI.00149-18 . Pubmed Central PMCID: 5952131. 29563291 PMC5952131

[66] Matumoto M. A note on some points of calculation method of LD50 by Reed and Muench. Jpn J Exp Med. 1949 Sep;20(2):175–9. . Epub 1949/09/01. 15396956

[67] Sanjana NE, Shalem O, Zhang F. Improved vectors and genome-wide libraries for CRISPR screening. Nat Methods. 2014 Aug;11(8):783–4. doi: 10.1038/nmeth.3047 . Pubmed Central PMCID: PMC4486245. Epub 2014/07/31. 25075903 PMC4486245

[68] Shalem O, Sanjana NE, Hartenian E, Shi X, Scott DA, Mikkelson T, et al. Genome-scale CRISPR-Cas9 knockout screening in human cells. Science. 2014 Jan 3;343(6166):84–7. doi: 10.1126/science.1247005 . Pubmed Central PMCID: PMC4089965. Epub 2013/12/18. 24336571 PMC4089965

[69] Meister G, Landthaler M, Patkaniowska A, Dorsett Y, Teng G, Tuschl T. Human Argonaute2 mediates RNA cleavage targeted by miRNAs and siRNAs. Mol Cell. 2004 Jul 23;15(2):185–97. doi: 10.1016/j.molcel.2004.07.007 . Epub 2004/07/21. 15260970

[70] Lei CQ, Wu X, Zhong X, Jiang L, Zhong B, Shu HB. USP19 Inhibits TNF-alpha- and IL-1beta-triggered NF-kappaB activation by deubiquitinating TAK1. J Immunol. 2019 Jul 1;203(1):259–68. doi: 10.4049/jimmunol.1900083 . Epub 2019/05/28. 31127032

