# Supplementary figures and images for: MicroRNA-200c-targeted contactin 1 facilitates the replication of influenza A virus by accelerating the degradation of MAVS
Source: PLoS Pathog. 2022 Feb 16;18(2):e1010299. doi: 10.1371/journal.ppat.1010299 (PMC8849533; doi:10.1371/journal.ppat.1010299)

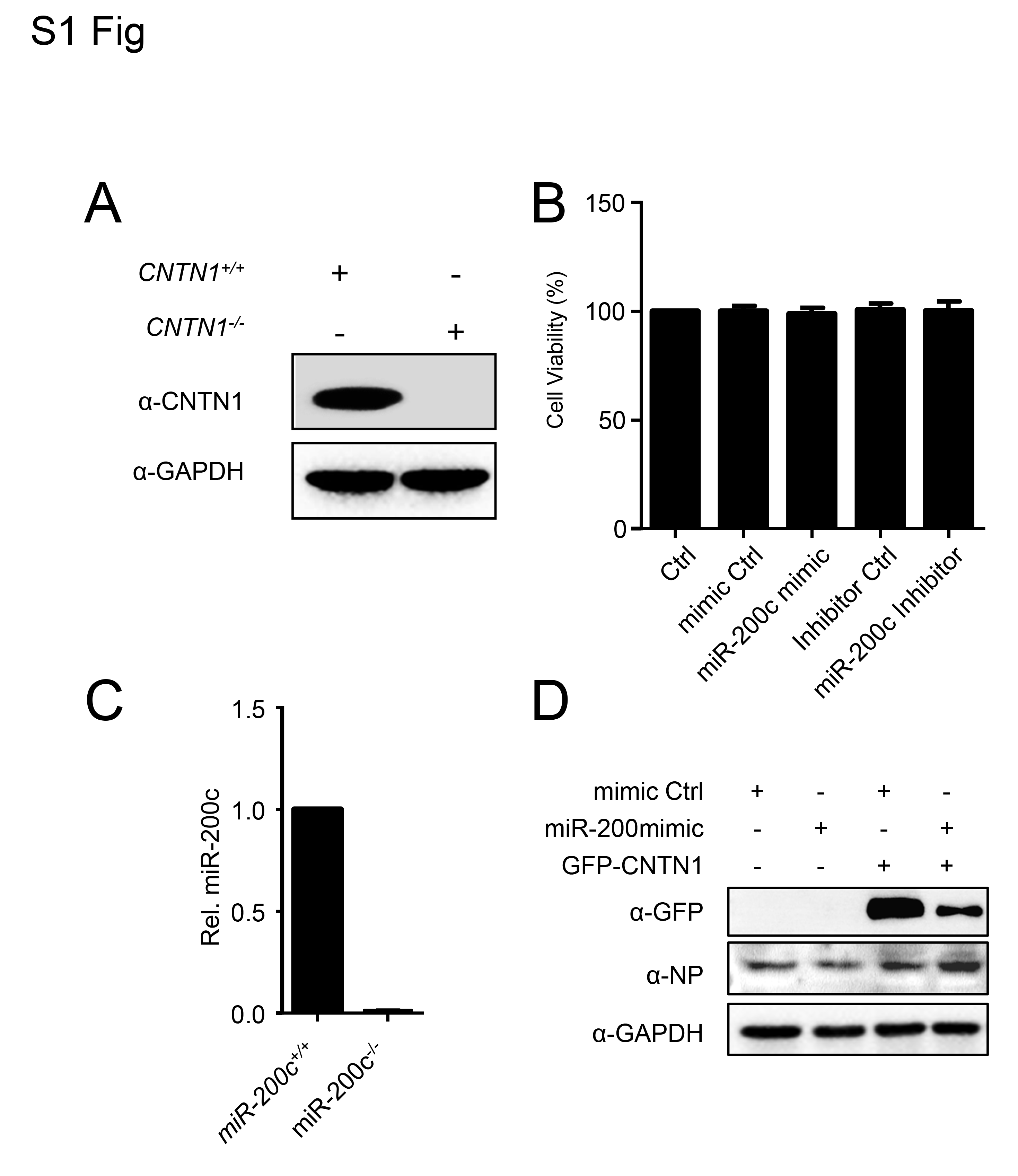

Supplement: S1 Fig — (A) The expression of CNTN1 in CNTN1+/+ and CNTN1-/- cells. (B) HEK293 cells were transfected with miR-200c mimic, mimic control, miR-200c inhibitor, or inhibitor control. Twenty-four hours later, cell viability was measured by CCK8. (C) The expression of miR-200c in miR-200c+/+ and miR-200c-/- cells. (D) A549 cells were transfected with the miR-200c mimic or mimic control, and the CNTN1 expression plasmid or Vec. Twenty-four hours later, the cells were infected with H5N6 virus at an MOI of 3. At the 12 hours post-infection, the cells were subjected to immunoblotting analysis with the indicated antibodies. (TIF) [file ppat.1010299.s001.tif]

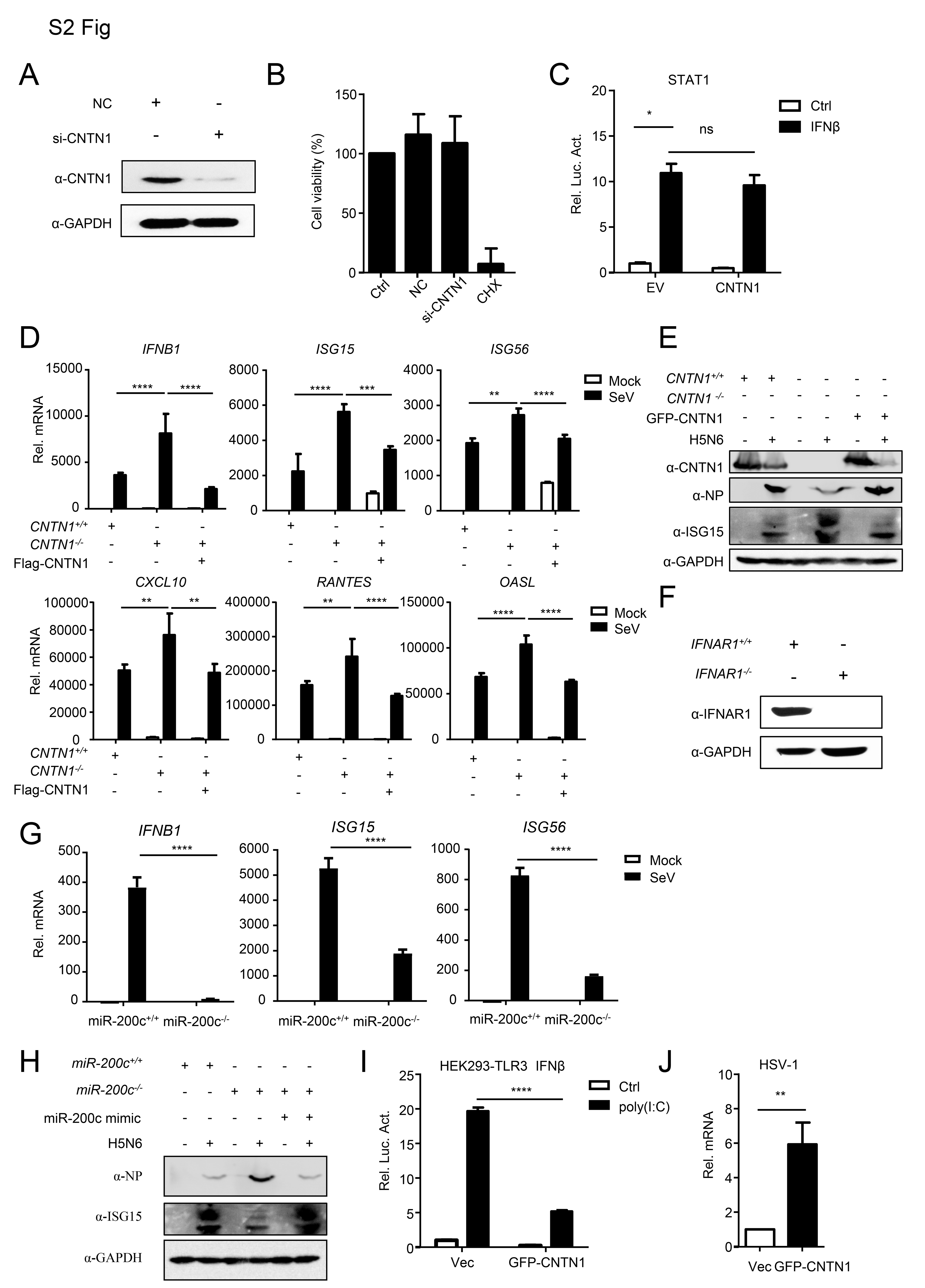

Supplement: S2 Fig — (A) The expression of CNTN1 in NC- or si-CNTN1-transfected cells. (B) A549 cells were transfected with CNTN1 siRNA or NC. At twenty-four hours after transfection, cell viability was measured by CCK8. (C) Luciferase reporter plasmids (STAT1-Luc) and pRL-TK plasmid were co-transfected into HEK293 cells, along with Flag-CNTN1 or Vec. At twenty-four hours after transfection, the cells were left untreated or were treated with IFN-β for 12 h before reporter assays. (D) CNTN1+/+ and CNTN1-/- cells were transfected with Flag-CNTN1 or Vec, and subsequently left uninfected or infected with SeV for 12 h before qPCR analysis. (E) CNTN1+/+ and CNTN1-/- cells were transfected with Flag-CNTN1 or Vec, and subsequently left uninfected or infected with H5N6 virus for 24 h before immunoblotting analysis. (F) The expression of IFNAR1 in IFNAR1+/+ and IFNAR1-/- cells. (G) miR-200c+/+ and miR-200c-/- cells were left uninfected or infected with SeV for 12 h before qPCR analysis. (H) miR-200c+/+ and miR-200c-/- cells were transfected with miR-200c mimic or mimic control, and subsequently left uninfected or infected with H5N6 virus for 12 h before immunoblotting analysis. (I) Effects of CNTN1 on TLR3-mediated signaling. 293-TLR3 cells were transfected with IFN-β reporter and pRL-TK plasmid along with the indicated plasmids for 18 h and then were treated or untreated with poly(I:C) (20 μg/mL) for 12 h before reporter assays. (J) A549 cells were transfected with either the CNTN1 expression plasmid or Vec. Twenty-four hours later, the cells were infected with HSV-1 virus. At 24 h post-infection, the expression of HSV-1 RNA was quantified using qPCR analysis. (TIF) [file ppat.1010299.s002.tif]

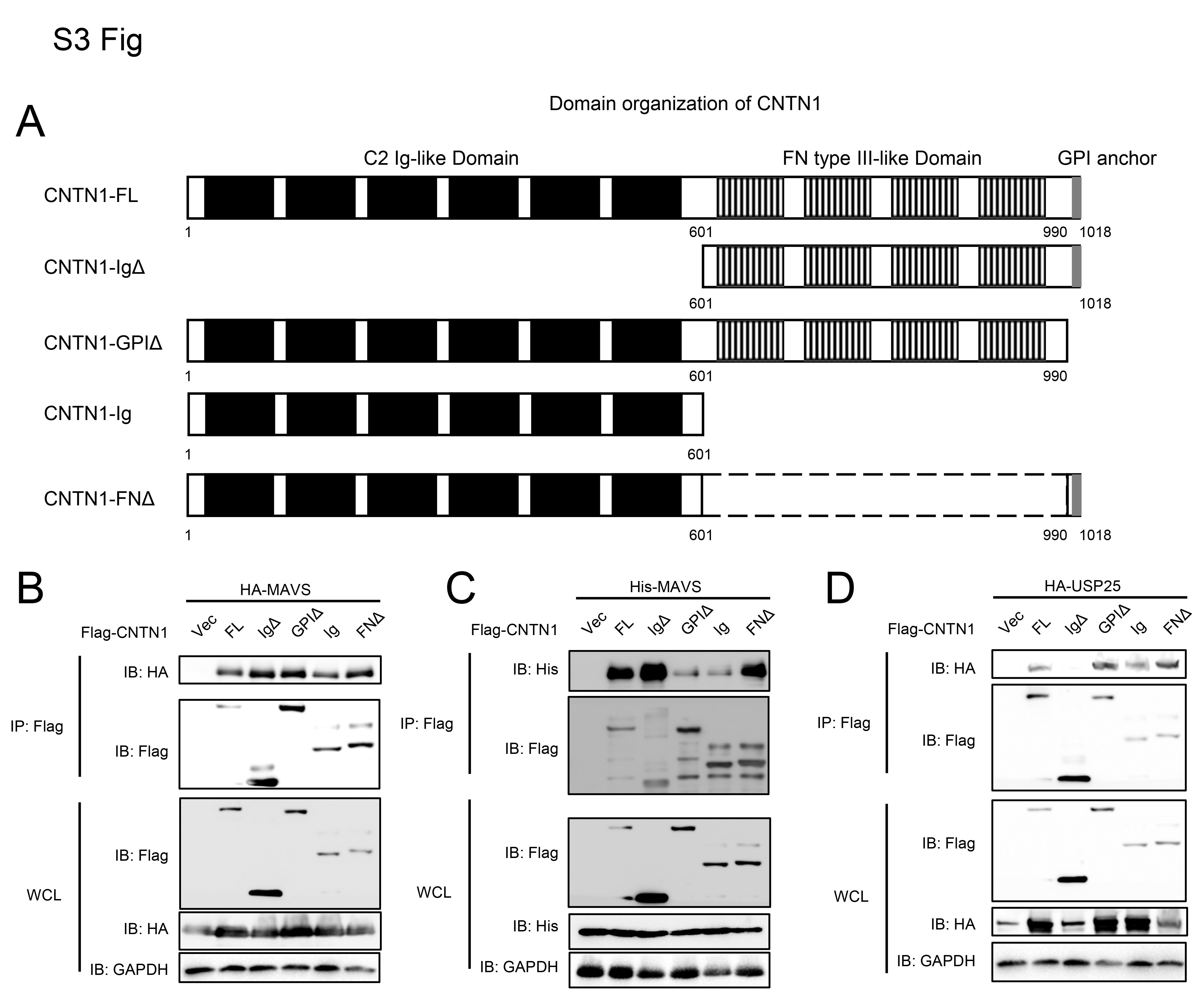

Supplement: S3 Fig — (A) Schematic representation of the domain organization of CNTN1. (B) CNTN1 interacts with MAVS. HEK293 cells were transfected with the indicated plasmids for 24 h. Then the co-immunoprecipitation and immunoblotting analysis were performed with the indicated antibodies. (C) CNTN1 interacts with purified His-MAVS. HEK293 cells were transfected with the indicated plasmids for 24 h. Then the cell lysate was mixed with purified His-MAVS, and the co-immunoprecipitation and immunoblotting analysis were performed with the indicated antibodies. (D) CNTN1 interacts with USP25. HEK293 cells were transfected with the indicated plasmids for 24 h. Then the co-immunoprecipitation and immunoblotting analysis were performed with the indicated antibodies. (TIF) [file ppat.1010299.s003.tif]

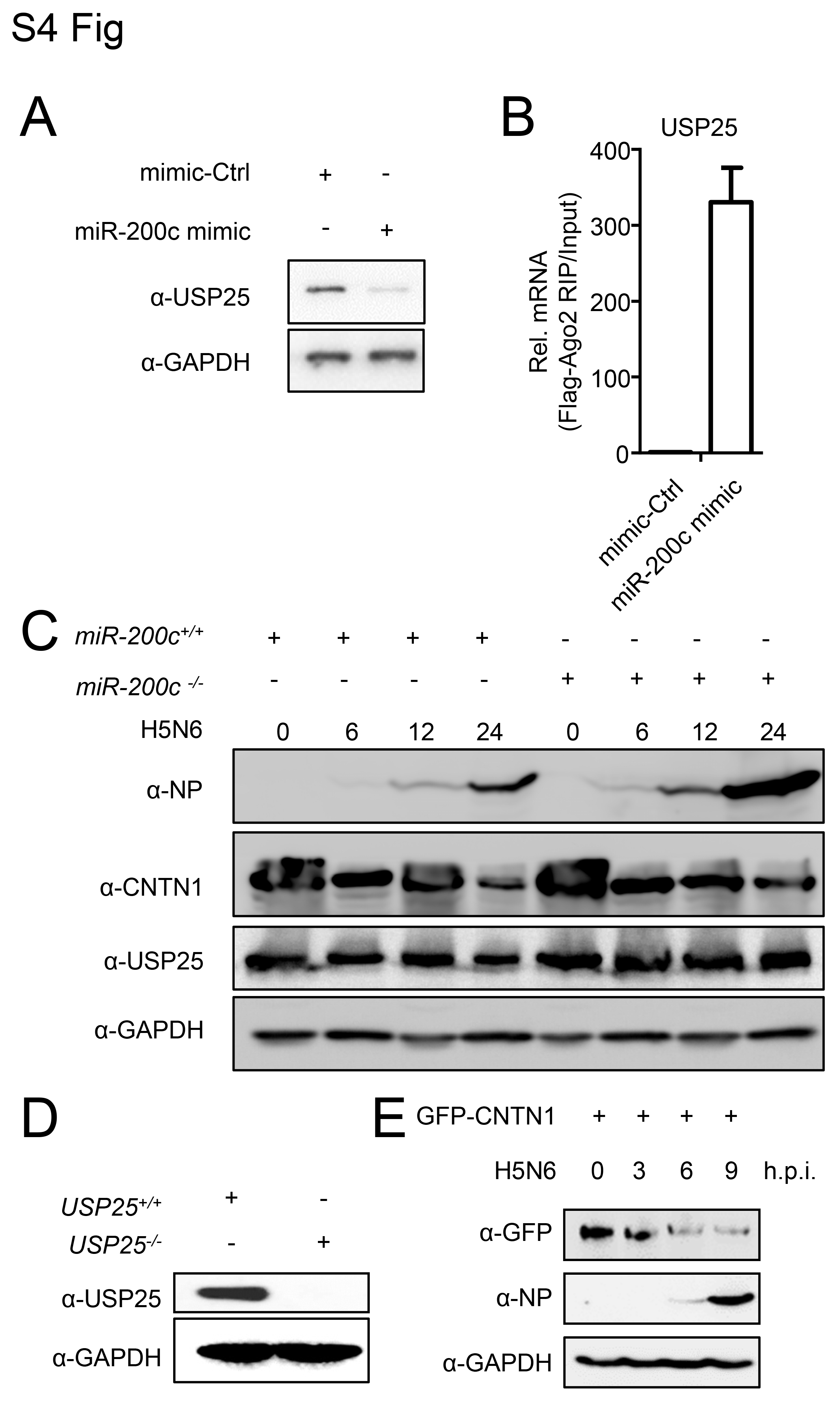

Supplement: S4 Fig — (A) HEK293 cells were transfected with miR-200c the mimic or mimic control for 24 h before immunoblotting analysis. (B) A549 cells were transfected with Flag-Ago2 in the presence of either the miR-200c mimic or mimic control. Twenty-four hours later, the cells were subjected to RIP assay with an anti-Flag antibody. The level of USP25 mRNA was quantified by qPCR. (C) miR-200c+/+ and miR-200c-/- cells were transfected with GFP-CNTN1 plasmid or Vec. Twenty-four hours later, the cells were infected with H5N6 virus (MOI = 1) for indicated times before immunoblotting analysis. (D) The expression of USP25 in USP25+/+ and USP25-/- cells. (E) A549 cells were transfected with GFP-CNTN1 plasmid. Twenty-four hours later, the cells were infected with H5N6 virus (MOI = 1) for indicated times post-infection. (TIF) [file ppat.1010299.s004.tif]
